# Discovery of New Ligand with Quinoline Scaffold as Potent Allosteric Inhibitor of HIV-1 and Its Copper Complexes as a Powerful Catalyst for the Synthesis of Chiral Benzimidazole Derivatives, and *in Silico* Anti-HIV-1 Studies

**DOI:** 10.1155/2023/2881582

**Published:** 2023-04-21

**Authors:** Sabikeh G. Azimi, Ghodsieh Bagherzade, Mohammad Reza Saberi, Zeinab Amiri Tehranizadeh

**Affiliations:** ^1^Department of Chemistry, Faculty of Sciences, University of Birjand, Birjand 97175-615, Iran; ^2^Department of Medical Chemistry, School of Pharmacy, Mashhad University of Medical Sciences, Mashhad 91775-1365, Iran

## Abstract

In this paper, the novel Schiff base ligand containing quinoline moiety and its novel copper chelate complexes were successfully prepared. The catalytic activity of the final complex in the organic reaction such as synthesis of chiral benzimidazoles and anti-HIV-1 activity of Schiff base ligand and the products of this reaction were investigated. In addition, green chemistry reactions using microwaves, powerful catalyst synthesis, green recovery and reusability, and separation of products with economic, safe, and clean methods (green chemistry) are among the advantages of this protocol. The potency of these compounds as anti-HIV-1 agents was investigated using molecular docking into integrase (IN) enzyme with code 1QS4 and the GROMACS software for molecular dynamics simulation. The final steps were evaluated in case of RMSD, RMSF, and Rg. The results revealed that the compound VII exhibit a good binding affinity to integrase (Δ*g* = −10.99 kcal/mol) during 100 ns simulation time, and the analysis of RMSD suggested that compound VII was stable in the binding site of integrase.

## 1. Introduction

AIDS (acquired immune deficiency syndrome) is a type of disease that is caused by the entry of the human immunodeficiency virus (HIV) which attacks the immune system.

HIV infects vital cells in the immune system, such as the helper T cells (specifically CD_4_^+^T cells), macrophages, and the dendritic cells [1, 2]. If the number of CD_4_^+^T cells count is below 200 cells per microliter, then it is diagnosed as AIDS which results in the weakening of the immunity system [3].

HIV is of two types HIV-1 and HIV-2. HIV-1, contains three enzymes in the HIV pol gene, namely, reverse transcriptase (RT), protease (PR), and integrase (IN) [4]. HIV-1 integrase (IN) is the principal enzyme that helps in the reproduction and entrance of the virus into the host cell DNA [5]. This enzyme has an active site that contains Mg^2+^. This element is an important cofactor for binding the virus to the host cells; thus, most of the inhibitory medicines chelate with the Mg cofactor to deactivate the integrase [6–12]. We designed and synthesized the novel Schiff base and its Cu complex as potent inhibitors of the integrase (IN).

The utilization of catalysts for expansion and synthesis of the novel structures is an efficient and interesting method in organic chemistry and especially, beneficial catalysts play an important role in the improvement of eco-friendly green methods in chemical reactions.

Among the heterocyclic scaffolds, benzimidazoles are the useful structural motif for the development of pharmaceutical molecules and have attracted a lot of attention from the chemists. These series of compounds have a significant place in biochemistry as they have a wide range of biological activities such as anticancer [11, 13, 14], antidiabetic [15], and antibacterial which has been well studied in compounds such as bisbenzimidazoles and 2-chloromethyl-1H-benzimidazoles. The benzimidazole-quinolone hybrid, 1-(benzimidazole-2-yl) urea derivative, and 2-arylbenzimidazole-5-carboxamide derivative target bacterial DNA gyrase and topoisomerase [16–19]. Recent surveys have revealed that benzimidazole derivatives have significant effects as antimicrobial agents. Benzimidazole derivatives were found to interact with DNA and kill or inhibit the growth of different microbial strains [20-22]. Also, these compounds have the properties of antituberculosis [23], antioxidant [24], anti-inflammatory [25], anticonvulsant [26], antitumor, and anthelmintic like drugs with 5,6-dinitro- and 2-trifluoromethyl benzimidazole derivatives [27], antiprotozoal like drugs 2-aminoazimitazole [28].

Another important class of nitrogen heterocycles with great therapeutic importance can be pointed out to quinoline derivatives. Some of the compounds containing quinoline nucleus proved to be a promising pharmacophore in generating small molecules with anticancer, antimicrobial, antimalarial, anti-inflammatory, neuroprotective, antiviral activities [30], and anti HIV-1 effects [31]. Another application of quinolines is working as catalysts, for example, effective synthetic methodology for the syn selective and enantioselective synthesis of biologically active imidazolines [32–36].

In the present study, we tried synthesizing a new Schiff base ligand with a quinoline scaffold (E-N-[2-(1H-Benzimidazole-2-yl) phenyl]-1-(2-chloro-3 -quinolinyl) methanimine, under solvent-free, microwave-assisted conditions to enhance the microwave effects [37] and to reduce environmental concerns, with anti-HIV properties. We then succeeded in the synthesis of a strong trinuclear copper (II) complex catalyst for the preparation of the chiral benzimidazoles with the aim of obtaining a medicinal enantiomer by inhibiting the HIV virus integrase enzyme and reducing drug side effects.

We used microwave radiation as an effective technique in the synthesis of benzimidazoles in accordance with green chemistry protocols to increase the efficiency and area and selectivity of stereo reactions, energy efficiency, and reduction of reaction cycle time. Due to the importance of these compounds, vital amino acids were used as organic components in the synthesis of the compounds of this project. In addition, the use of the enantiomerically pure form of L-amino acids also provided the possibility of investigating the chiral structure. The amount of photorotation of chiral benzimidazole derivatives and the effect of using microwave radiation on their stereochemistry were also investigated.

Also, insilico studies of these compounds as anti-HIV-1 agents with molecular docking studies using the MOE program to find the best orientation of the ligand with the lowest energy in the active site of the protein on the integrase enzyme with the code 1QS4 and molecular dynamics simulation studies using the GROMACS software was analyzed for 100 ns.. After the completion of the simulation, structural changes, stability, and flexibility of the system were also evaluated by calculating RMSD, RMSF, and Rg.

## 2. Materials and Methods

### 2.1. Material

All reagents and solvents were purchased from Merck and Sigma-Aldrich and used without further purification. The progress of all reactions was monitored by thin-layer chromatography with 0.25 mm Silica gel plates (60 GF-254, Merck Co, Darmstadt, Germany) and visualized using fluorescent and UV light.

We also used a synthetic microwave to perform a series of reactions (Milestone Srl model; Microsynth, Italy, 2500 VA F02D 2450 MHz). Melting points were measured with an electrothermal melting point apparatus (Stafford, UK). Infrared spectra were recorded on a Perkin Elmer Model 1420 spectrometer (KBr disks, Massachusetts, USA). ^1^H NMR and ^13^CNMR spectra were determined at 300 MHz, respectively (DMSO-d6 and CDCl3-d6, TMS) on a Bruker FT-300 MHz instrument (Karlsruhe, Germany) [38]. The mass spectrum was obtained from a mass spectrometer model: Varian CH7. Inductively coupled plasma (ICP) spectroscopy was performed by an OPTIMA 7300DV device. Energy-dispersive X-ray spectroscopy (EDX) analysis was performed using field emission scanning electron microscope, model: LMU TESCAN BRNO-MIRA3. CHNS elemental analysis device model: Costech 4010 was used for elemental analysis. Optical rotations were evaluated with a polarimeter model Polax-2L-ATAGO in DMSO with a 10 cm cell.

### 2.2. Methods

#### 2.2.1. Chemistry

**Scheme 1:**
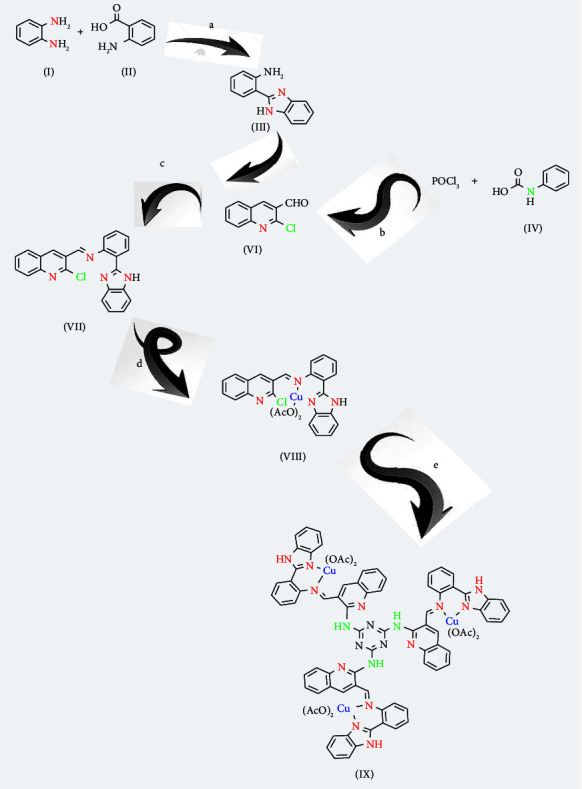
Reagents and conditions: (a) MW and PPA, (b) dry DMF and reflux, (c) DBU, r.t, and dry ethanol; (d) copper (II) acetate, DMSO, and reflux, and (e) melamine, DMF, K_2_CO_3_, and reflux.

The synthetic routes to target compounds containing the quinoline ring are illustrated in Scheme 1. Schiff base ligand (E)-N-[2-(1H-Benzimidazole-2-yl) phenyl]-1-(2-chloro-3-quinolinyl) methenamine (III) was synthesised through the reaction of the 2-(2-Aminophenyl)-1H-benzimidazole (І) with the 2-Chloro-3-quinolinecarbaldehyde (II) in the presence of a catalytic amount of 1,8-Diazabicyclo (5.4.0) undec-7-ene (DBU) in dry ethanol solvent at room temperature. Then, copper (II) complex was performed with high efficiency by reaction of the Schiff base ligand with copper (II) acetate solution in DMSO. Reaction of the copper (II) complex with melamine in presence of potassium carbonate in DMF led to the formation of trinuclear cationic copper (II) complex (Scheme 1). The chemical structures of the final compounds were characterized by ^1^H NMR, ^13^C NMR, IR, and MS spectroscopy, and by elemental analysis such as inductively coupled plasma spectrometry (ICP), energy-dispersiveX-ray spectroscopy (EDX), and CHN.

#### 2.2.2. Molecular Docking

Docking is a computational technique that can predict the interaction between two molecules. In principle, molecular docking studies were used to find the best orientation of a ligand with the lowest energy in the protein active site which represents a strong affinity of the substrate towards the enzyme. One of the software used for docking of the ligand into the receptor was MOE 2019. The PDB file for the ligands and the integrase protein (PDB ID: 1QS4) for docking studies was taken from the RCSB PDB. According to the tutorial of MOE 2019 [39], in the next step, extra chains of the protein have been removed by the MOE program. Then, by means of the quick prep panel command, polar hydrogens were added, the structural defects were eliminated, and the energy minimization process was applied. In order to dock in the active site of the enzyme, which contains Mg^2+^, the triangle matcher method was set to find the best placement. For the scoring matrix the London ΔG algorithm was chosen, and finally, the best 100 poses were set. After the refinement of the results, the top 30 results were sorted out based on the lowest Δ*G*. The best of the docking result was selected according to the orientation of the ligand in the active site and also the minimum Δ*G* energy obtained from its interaction with the receptor.

#### 2.2.3. Molecular Dynamic Simulation

Molecular dynamic simulation was performed to elucidate the behavior of the enzyme upon binding to its inhibitor and to maintain the stability of the system through interaction with ions and water during the simulation. The molecular dynamics (MD) program GROMACS [40, 41] was used to accomplish the MD simulations. By using GROMACS software (version 2019) and with the CHARMM36 force field a topology file (Topol. top) of protein was created and the topology file of the ligands was obtained from SwissParam [42].

The MD simulations is performed in a virtual cubic box which is filled with explicit water model (SPC/E). The protein is located in the center of the box and the distance from the neighboring box was set to one nanometer.

In each simulation, the system was neutralized by means of ions, and all the system was equilibrated at the temperature of 310.15 K with the NPT ensemble. Finally, MD simulations were performed at 100 ns. Visual Molecular Dynamics (VMD) and PyMOL software were used to analyze the results of the observations.

In this study, structural changes, stability, and flexibility of the system were monitored by calculating the values of root mean square deviation (RMSD), root mean square fluctuation (RMSF), radius gyration (Rg), the number of hydrogen bonds.

## 3. Results and Discussion

### 3.1. EI-MS Electronic Spectra

**Figure 1:**
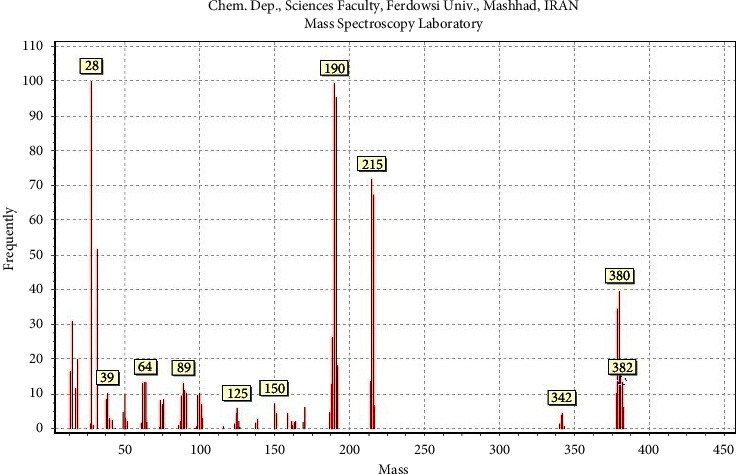
EI-MS electronic spectra images of compound VII.

The molecular ion peak of free ligand at 382 m/z and the main fragments at *m*/*z* (%) 382 (15), 380 (40), 215 (73), 190 (99), 28 (100), and 90 (12) were obtained by the use of mass spectrometry (Figure 1).

### 3.2. FT-IR Electronic Spectra

**Figure 2:**
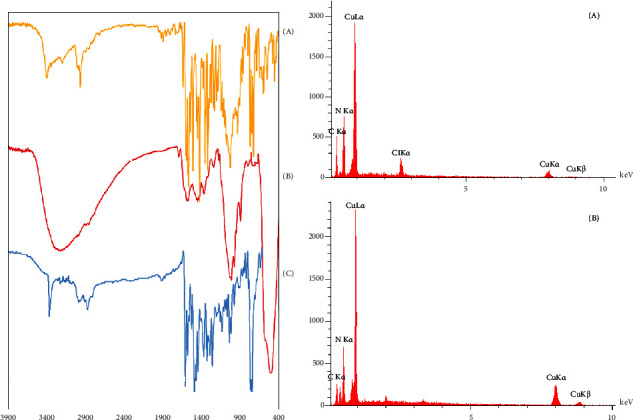
FT-IR spectrum. (a) Images of (A) compound VIII, (B) compound IX, and (C) compound VII. EDX spectra (b) images of (A) compound VIII and (B) compound IX.

According to Figure 2, the FTIR spectrum of the synthetic Schiff base ligand displayed characteristic peaks at the points of 736 cm^−1^ (C-Cl stretching), 1377 cm^−1^ (C-N stretching), 1499 cm^−1^, and 1589 cm^−1^ (C=C aromatic stretching vibration), as well as sharp absorption peaks was observed around the regions of 1616 cm^−1^ (C=N stretching vibration), 2880 cm^−1^ (C-H of CH=N stretching vibration), 3150 cm^−1^ (C-H aromatic stretching vibration), and 3417 cm^−1^ (N-H aromatic stretching vibration).

Subsequent to the complexation, the redshift in (-C=N-) of Schiff base ligand generated the peak for imine bond (1616 cm^−1^), which shifted towards wave numbers by about 11 cm^−1^ (1627 cm^−1^). This observation indicated the ligand coordination of nitrogen to the metal atom and expectedly, it increased the electron density of azomethine link and caused a shift in the C=N band. Moreover, the appearance of two absorption bands at 1446 and 1662 cm^−1^ was due to the presence of symmetric and asymmetric stretching of coordinated acetate. Extensive infrared spectral studies reported on metal acetate complexes, indicating the coordination of acetate ligand to the metal center in either a monodentate, bi-dentate, or bridging manner. The synergistic effect of adding melamine to synthetic copper complex, which is recorded as peak(c), resulted in losing and changing the peak of chlorine bond in the appearance, flattening, and displacement of peaks. Also, the absorption bands at 1051 and 1620 cm^−1^ could be assigned to the C-N and C=N melamine stretching, respectively (Figure 2(b)).

### 3.3. Elemental Analysis (CHN, EDX, and ICP)

According to the elemental analysis (CHN, EDX, and ICP) of compound VII, compound VIII, and compound IX, the results of EDX analysis displayed the presence of C, N, Cl, and Cu elements and the disappearance of chlorine peak in compound IX, which can be considered as an acceptable evidence for compound VIII and compound IX (Figure 2). The outcomes of ICP and EDX showed the amount of existing copper in compound IX to be three times more than that of the compound VIII, while confirming the proposed approximated spatial structure for compound IX.

### 3.4. Preparation of the Chiral Benzimidazole Derivatives with Compound IX as the Catalyst

**Scheme 2:**
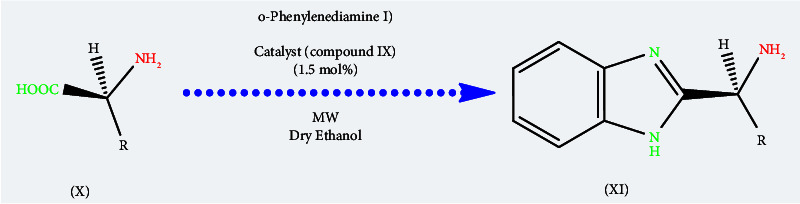
Overview of the synthesis method of the chiral benzimidazole derivatives with trinuclear cationic copper (II) complex (compound IX).

Compound IX was used as the catalyst in optimal conditions to perform the synthesis of chiral benzimidazoles XI by the means of different natural L-amino acids X and o-phenylenediamine. The synthesis path of these benzimidazoles is shown in the Scheme 2. Almost every substrate was capable of obtaining the satisfactory yields of products (basically over 66 %). The results of one-pot microwave-assisted method displayed some apparent advantages including higher yield, shorter reaction time, less reagent consumption, and simpler reaction equipment. Other advantages of this method include the synthesis of new benzimidazoles (compounds f and h)

Due to the importance of stereochemistry, optical rotations of chiral benzimidazole derivatives synthesized using natural L-amino acids were also investigated. The effects of this microwave-assisted method on stereochemistry were investigated by experiments and the stereochemistry of chiral L-amino acids was not affected under the reaction conditions.

Based on the studies and comparisons presented in Table 1, trinuclear copper catalyst was used in the synthesis reaction of benzimidazoles as an effective catalyst with high recovery and durability in green conditions. The efficiency of trinuclear catalysts was compared with the previous systems and it was found that the synthetic catalyst has a high catalytic efficiency in addition to its reusability and easy recovery. The ability to recover the catalyst after separation by centrifugation in each experiment was evaluated by measuring the reaction efficiency in several steps, and no significant change was observed.

### 3.5. Molecular Docking Studies

The integrase of a 32 kDa protein consists of three structural domains including (1) amino-terminal domain, (2) catalytic nucleus domain (CCD), and (3) terminal carboxy domain (CTD). Although the role of every three domain is essential for integration, yet the amino-terminal domain is particularly crucial for the case of zinc, while manganese (Mn^2+^) and magnesium (Mg^2+^) are bounded to CCD as the active site of enzyme that is composed of acidic residues (i.e., Asp64, Asp116, and Glu152).

CCD consists of 50 to 212 amino acids and is recognized as the major domain of IN. It is known as a catalytic domain due to the its participation in catalytic reactions. Raltegravir (Isentress) is the first FDA-approved medicine for HIV that is based on inhibiting the HIV integrase. Furthermore, structural studies were performed due to the importance of molecule geometry in causing inhibitory effects.

**Figure 3:**
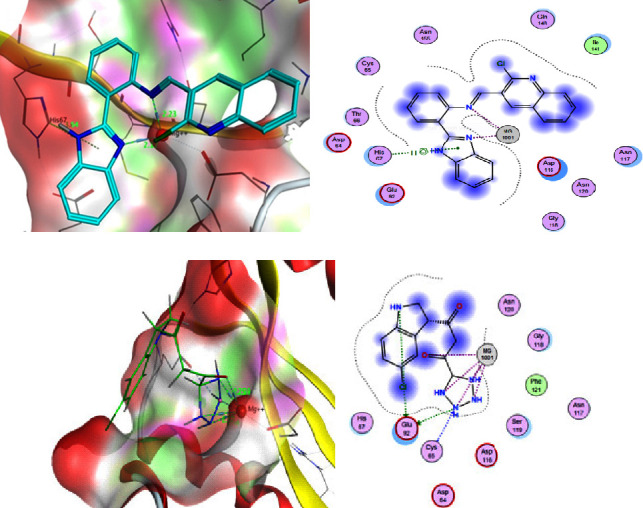
(a) 3D representation of the docking result of compound VII into the tunnel-like binding site of HIV-1 integrase CCD dimer. (b) 2D representation of the docking result of compound VII into the tunnel-like binding site of HIV-1 integrase CCD dimer. (c) 3D representation of the docking result of 5ClTEP into the tunnel-like binding site of HIV-1 integrase CCD dimer. (d) 2D representation of the docking result of 5ClTEP into the tunnel-like binding site of HIV-1 integrase CCD dimer.

According to Figures 3(c) and 3(d), the docking of reference structure (cocrystal (1-(5-chloroindol-3-yl)-3-hydroxy-3-(2H-tetrazol-5-yl)-propenone (5ClTEP)) with integrase in the active site led to the chelating of tetrazole ring and the carbonyl group with Mg^2+^ ions, which is comparable to the docking results of compound VII, benzimidazole nitrogen, and imine with Mg^2+^ ion chelating (Figures 3(a) and 3(b)). Benzimidazole also improved the antiintegrase and antiviral potency of the scaffold [49–51].

The best pose of the compound VII obtained from docking study in the active site of integrase is shown in Figures 3(a) and 3(b). It was seen that Mg^2+^ ion was pentacoordinated with Asp116 and Asp64 as well as with the nitrogen of the imine bond and the nitrogen of the benzimidazole ring of compound VII.

Compound VII with the lowest binding energy of −10.99 kcal/mol has a better result compared to the docking result of cocrystal structure of the 5ClTEP with the lowest binding energy of −9.55 kcal/mol in the cavity of the active site which contains polar amino acids. Also, there was *π* − *π* stacking interaction with the imidazole group of His67 at a distance of 3.64 Å, which improved the binding affinity of the compound VII with integrase.

**Figure 4:**
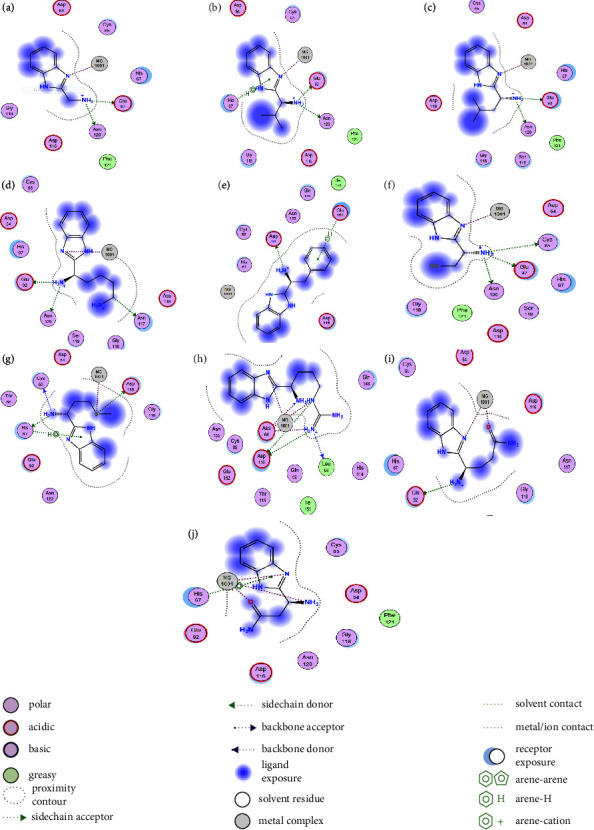
2D representation of the interaction of HIV-1 integrase CCD dimer with compounds. (a) Compound (a). (b) Compound (b). (c) Compound (c). (d) Compound (d). (e) Compound (e). (f) Compound (f). (g) Compound (g). (h) Compound (h). (i) Compound (i). (j) Compound (j).

**Figure 5:**
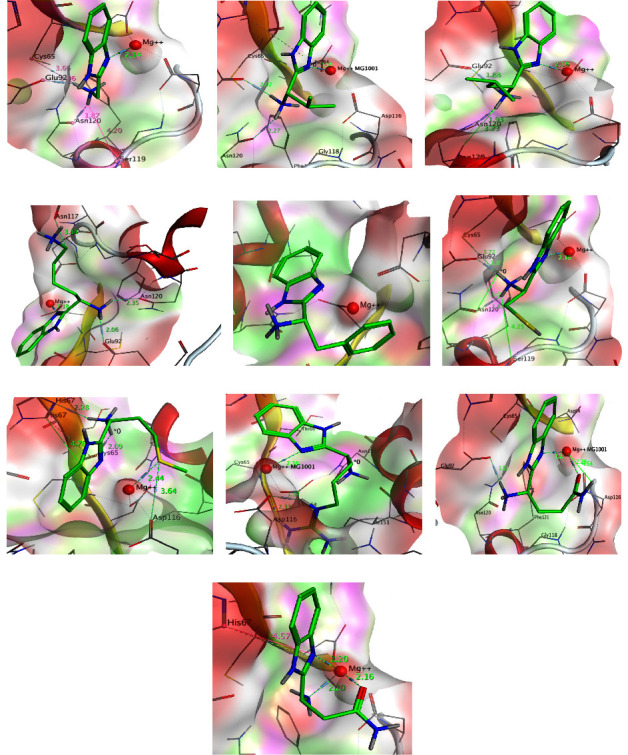
Close-up view of the binding interactions of HIV-1 integrase CCD dimer with compounds (a) Compound (a). (b) Compound (b). (c) Compound (c). (d) Compound (d). (e) Compound (e). (f) Compound (f). (g) Compound (g). (h) Compound (h). (i) Compound (i). (j) Compound (j). Interacting residues of HIV-1 integrase CCD dimer are shown in sticks colored by atom type, carbon is colored green, nitrogen is colored blue, oxygen is colored red, and Mg^2+^ is colored red.

Docking interaction analysis of the integrase with compounds A-J is shown in Figure4. The best pose of each compound in terms of free energy of binding was extracted from the 100 generated top poses of the binding mode. All the docked compounds showed binding energy ranging from −6.98 to −10.99 kcal/mol. Benzimidazole derivatives docked well into the integrase. Integrase is a magnesium dependent enzyme and its active site consists of a long and narrow tunnel leading to a cavity that contains the catalytic Mg^+2^ ion. The study revealed that compound (j) exhibited the best inhibitory potential against the enzyme. Compound (j) showed the hydrogen bonding and the electrostatic and hydrophobic interactions with the active site residues of the integrase (Figure 5).

Mg ion was pentacoordinated with Asp116 and Asp64 as well as with the carbonyl, amine, and the nitrogen of the benzimidazole ring of compound (j). There was also *π*-*π* stacking interaction with the benzyl group of His67 at a distance of 4.57 Å, which improved the binding affinity of compound (j) to integrase.

### 3.6. Molecular Dynamic Simulation

#### 3.6.1. Root Mean Square Deviation (RMSD)

Generally, in an MD simulation, the RMSD value is used for analyzing the stability of protein and for predicting the structural changes in protein. RMSD values depend on the binding interaction and energy between the protein and the ligand. The RMSD value is usually used to validate the connection protocol. If the binding protocol is able to create a similar binding state of the ligand due to the biological configuration of the same ligand in the complex crystal structure of the protein, it means that the binding is confirmed and also RMSD information about the deviation from the original structure and the stability of the third structure of the enzyme is confirmed.

**Figure 6:**
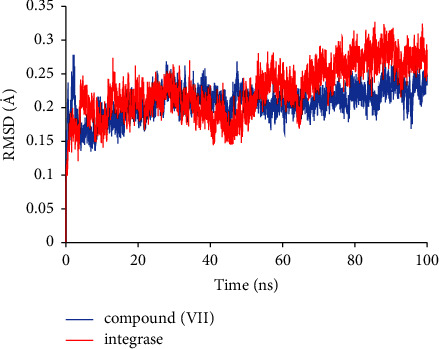
RMSD of alpha carbon of the integrase are shown as function of the simulation time (100 ns) at 300 K and the overlayed RMSD plots are integrased in red and compound VII in blue.

According to Figure 6, the deviation of alpha carbon from the standard structure for integrase alone was equivalent (0.22 ± 0.038 nm). In the presence of compound VII from 60 ns onwards, its stability increased slightly. The average RMSD value in the presence of the compound VII also converged very close to integrase with value of 0.20 ± 0.021 nm. RMSD in all cases has a relative stability, especially in the presence of compound (VII) led to greater stability at about 0.05 nm in RMSD especially after 49 nano seconds onwards.

**Figure 7:**
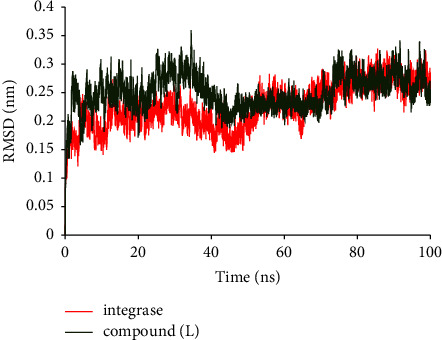
Plot of RMSD alpha carbon integrase during 100 ns of simulation in connection with compound (l) for integrase in red and for integrase with compound (L) in green.

RMSD plot in Figure 7 shows that the protein-ligand acquired equilibrium and the system was found to be stable especially after 50 ns till the end of the molecular dynamics simulation. Based on the analysis of RMSD of the protein backbone, a low RMSD value (RMSD∼0.15–0.3 nm) indicates high stability of the protein after interaction with the compound (j). The substrates could adequately form strong bonds based on the hydrogen bonding distance (<0.35 nm) into integrase active site. The average RMSD value of integrase was 0.22 ± 0.038 nm, whereas the average RMSD value of integrase in the presence of the compound (j) also converged very close to integrase with average RMSD value of 0.15 ± 0.037 nm.

**Figure 8:**
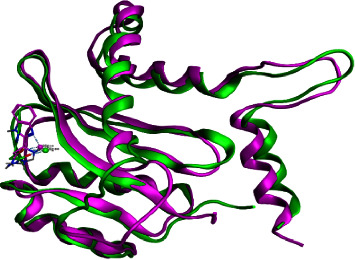
Close-up view of the binding interactions of compound (j) with HIV-1 integrase CCD dimer. Colored purple is before MD simulation and colored green is after MD simulation.

According to Figure 8, compound (j) remains in the active site after MD simulation and binds to Mg^2+^, amino acids Glu92, and Asn117, leading to the maximum stability of the protein.

#### 3.6.2. Root Mean Square Fluctuation (RMSF)

The ligand binding poses, energy, and interaction depend entirely on the residual fluctuation values (RMSF). The RMSF plot shows the fluctuation ratio at the residual level and is important for predicting the structural stability of proteins.

**Figure 9:**
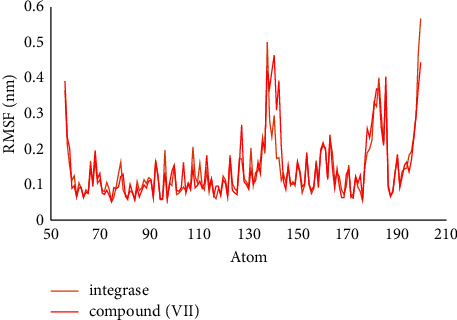
Graph of RMSF changes of alpha carbon integrase according to residue in connection with compound VII at 100 ns and 300 K. RMSF of integrase is in orange, and in the presence of compound VII, it is in red.

The high RMSF indicates higher degree of action. Conversely, low RMSF represents a more stable structure that only restricts motion during the simulation. [52] Residue occurs [53] as can be seen in the RMSF plot (Figure 9), and the mean RMSF values for the integrase are 0.14 ± 0.085 nm, and in the presence of compound VII, the values obtained are 0.14 ± 0.091 nm. The exceptionally low RMSF values for compound VII implicated high levels of structural stability, which explained the relatively stronger binding of compound VII with the active site residues that led to their lower flexibility.

As can be seen in Figure 9, high fluctuating regions of the integrase in interaction with compound VII include residues 142–148 located in the loops of protein that led to the change in the secondary structure of protein.

Results revealed that compound VII was stable and well-fitted in the binding site forming a static and stable protein-ligand complex.

**Figure 10:**
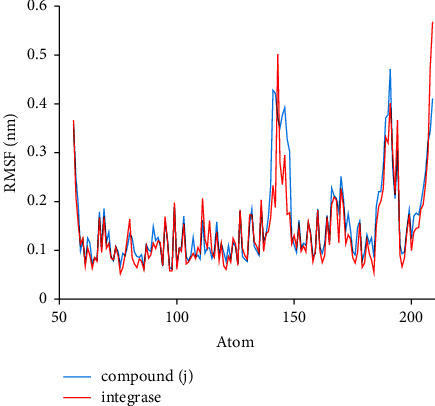
Plot of RMSF changes of alpha carbon integrase according to residue in connection with compound (j) for integrase in red and for integrase in the presence of compound (j) in blue.

As you can see in the RMSF plots in (Figure 10), the average RMSF values were calculated for the integrase enzyme (0.14 ± 0.085 nm) and in the presence of compound (j) the average RMSF values were calculated (0.15 ± 0.088 nm).

The exceptionally low values of RMSF for compound (j) are associated with high levels of structural stability, indicating a relatively stronger binding of the ligand to the active site residues, resulting in less flexibility. The high fluctuating residues indicate that they are far from the active site in protein rings or terminals.

The result showed that RMSF was stable and well-fitted in the binding site of the ligand forming a static and stable protein-ligand complex.

#### 3.6.3. Radius of Gyration (Rg)

The radius of rotation (Rg), which is used to describe the equilibrium composition of a total system, indicates the compactness of the protein structure, in other words, the root-square weight average of a group of atoms from their common center of mass.

**Figure 11:**
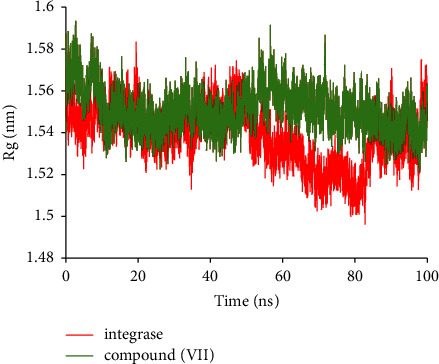
Plot of changes in Rg of alpha carbon integrase during 100 ns of simulation in connection with compound VII for integrase in red and for integrase in the presence of compound VII in green.

We also performed Rg analysis to observe the structural changes and dynamic stability of the ligand structures. The average calculated value of Rg from the trajectory integrase was 1.53 ± 0.013 nm, and in the presence of compound VII, it was 1.55 ± 0.010 nm, and the results are shown in Figure 11. The highest Rg plot proposes a looser packing of the amino acids and vice versa when the Rg value is at the lowest indicating a high compact conformation of the ligand complex.

As can be seen in Figure 11, the radius of gyrations for the first 10 ns was unchanged in the radius, but from 10 ns to 25 ns the radius in the ligand and especially in compound VII graph decreased which shows more protein compression. Maximum changes were observed for the radius of gyrations from 50ns to 90ns for integrase, indicating a decrease in compaction for integrase in the presence of compound (VII).

**Figure 12:**
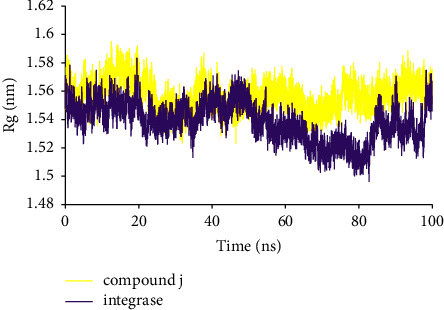
Plot of Rg changes of alpha carbon integrase during 100 ns of simulation in connection with compound (j) for integrase in purple and for integrase in the presence of compound (j) in yellow.

We performed Rg analysis to observe the conformational alterations and dynamic stability of the compound (j). The average calculated value of Rg from the trajectory integrase and ligand-bond protein was 1.53 ± 0.013 nm and 1.55 ± 0.010 nm, respectively which is shown in (Figure 12). The graphs of integrase , the radius of gyrations for the first 50 ns shows relative stability but at the 50 ns to the rest the graphs show fluctuation about 0.05 nm. The radius of gyrations of the integrase in the presence of compound (j) reached more stability.

Hydrogen bonds play an essential role in the stability of protein. This stability of the hydrogen-bond network formed by the ligand in the protein active site was analyzed which increased the binding affinity and stabilization of the ligand with integrase.

## 4. Green Chemistry Metrics Study

**Figure 13:**
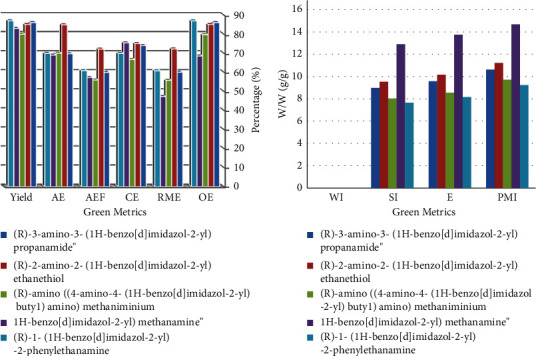
Green metrics including (a) AE, AEf, CE, RME, and OE. (b) PMI, E, SI, and WI for synthesis of the chiral benzimidazole derivatives with trinuclear cationic copper (II) complex as the catalyst.

Finally, a series of green metrics [54, 55] such as atom economy (AE), atom efficiency (AEF), carbon efficiency (CE), reaction mass efficiency (RME), optimum efficiency (OE), process mass intensity (PMI), E factor (E), solvent intensity (SI), and water intensity (WI) were calculated to evaluate the greenness of the synthesis of the chiral benzimidazole derivatives (Figure 13, See supporting information (SI) file for detailed calculations). High AE, AEF, CE, RME, and OE values for the synthesis of (R)-1-(1H-benzo[d]imidazol-2-yl)-2-phenylethanamine, (R)-2-amino-2-(1H-benzo[d]imidazole-2-yl) ethanethiol, 2-(2-Aminophenyl)-1H-benzimidazole, and (E)-N-[2-(1H-Benzimidazol-2-yl) phenyl]-1-(2-chloro-3-quinolinyl) methanimine show the greenness of the process well (See SI file for detailed calculations) (Figure 13(a)). The lower the PMI, E, and SI, the more favorable the trend is due to green chemistry. These values used in the synthesis of (R)-1-(1H-benzo[d]imidazol-2-yl)-2-phenylethanamine and 2-(2-Aminophenyl)-1H-benzimidazole and (E)-N-[2-(1H-Benzimidazol-2-yl) phenyl]-1-(2-chloro-3-quinolinyl) methanimine are less than 10 (Figure 13(b)). Hence, it can be concluded that in the case of high values of RME and low values of PMI, E, SI, and WI, the synthesis process of benzimidazoles is an efficient and green protocol. (See supporting information file (SIF) for detailed calculations).

## 5. Experimental Section

After the investigations, from the reaction of 2-(2-aminophenyl)-1H-benzimidazole (compound (I)) and 2-chloro-3-quinolinecarbaldehyde (compound (II)) in the presence of DBU in dry ethanol solvent at room temperature, a new Schiff base ligand ((E)-N-[2-(1H-Benzimidazole-2-yl) phenyl]-1-(2-chloro-3-quinolinyl) methanimine (compound (III)) was synthesized. Then succeeded in synthesis of the new copper (II) complex and a trinuclear cationic copper (II) complex from reaction of the new Schiff base ligand (compound (ІІІ)) and Cu (OAc)_2_ H_2_O.

### 5.1. General Procedure for the Synthesis of 2-(2-Aminophenyl)-1H-benzimidazole IIІ

In a 25-mLround-bottom flask, 3 mmol of o-Phenylenediamine I and 3 mmol of anthranilic acid II and 5 g polyphosphoric acid (PPA) were added and mixed with a mechanical mixer until a uniform paste was obtained. The reaction mixture was reacted with the microwave-assisted and solvent free green chemistry with an output power of 1000 W for 12 min at 160°C. After the reaction, the reaction vessel was cooled to room temperature and neutralized with ice-cold 10% aqueous sodium carbonate until gray deposits were obtained and were washed several times with distilled water and dried at room temperature. The separation of the products with this economic, safe, and clean method of green chemistry is one of the most important advantages of this reaction. The final product was recrystallized with aqueous ethanol (70%) and silver needle-shaped crystals were obtained. (97%) (Scheme 1).

### 5.2. General Procedure for the Synthesis of 2-Chloro-3-Quinolinecarbaldehyde V*І*

Dry DMF (15 mmol) was added to acetanilide (5 mmol) until mixed homogeneously in ice bath. Then, 5.56 ml and 6 mmol of phosphorylchloride V was added drop wise to the solution obtained in the previous step and stirred constantly for 20 min. Finally, the reaction mixture was refluxed for 16 h. After the reaction, the resulting product was cooled to room temperature and it was then transferred to a stirring ice container until a yellowish solid product was obtained. To increase the efficiency of the reaction, the pH of the resulting solution was regulated by the NaOH 50% to 14 and then the solution was stirred for 1 h. The sediments were filtered and washed with distilled water, and then was placed in an oven at 80°C for 5 h. The pure yellow crystals were gained by recrystallization from acetonitrile (m.p.147°C, 94% isolated yield) (Scheme 1).

### 5.3. General Procedure for the Synthesis of (E)-N-[2-(1H-Benzimidazol-2-Yl) phenyl]-1-(2-Chloro-3-Quinolinyl) Methanimine VII

The desired Schiff base ligand was performed with high efficiency by reaction of the 2-Chloro-3-quinolinecarbaldehyde (5mmol) and 2-(2-Aminophenyl)-1H-benzimidazole (5mmol) at room temperature in the presence of the 1, 8- diazabicyclo (5.4.0) undec-7-ene (DBU) (20μl) in dry ethanol. After completing the reaction, the final products were centrifuged and washed with ethanol and acetone until collected high purity white solid powder and the pure yellow crystals were gained by recrystallization from DMSO (m.p. 295–297°C, 74% isolated yield) (Scheme 1).

### 5.4. Loading with Cu (OAc)_2_

For the synthesis of compound VIII, copper ion was used as this ion is required by the body and is safe. So, first copper (II) acetate solution dissolved in DMSO was added drop wise to the reaction vessel containing the ligand in DMSO. Then, it was refluxed for 30 min and the dark brown powder obtained was separated by centrifuge and washed with distilled water, and dried at 80°C in an oven. Copper loading of compound VІІ was determined by the energy-dispersiveX-ray spectroscopy (EDX) and IR and CHN techniques. CHN analyses suggested a molecular formula of compound VIIІ (calc (%): C, 57.45; N, 9.93; H, 3.75), which reasonably agrees with the real amounts (%) of C (56.71), N (10.08), and H (3.24) (Scheme 1).

### 5.5. General Procedure for the Synthesis of Melamine-Functionalized Trinuclear Cationic Copper (II) Complex (Compound ІX)

At this stage, first melamine was weighed and transferred to a round bottom flask containing DMF (5 mL) and K_2_CO_3_ (3.0 mmol). Then, using the molar ratio of 3 : 1, some of the compound VIIІ: melamine and compound VIIІ was weighed and added to the reaction mixture. Then, the reaction mixture refluxed for 24 h. After the reaction completion, the desired product was removed with filtration and was washed with DMF, and finally was placed in a vacuum oven at 60°C for 12 h. Scheme 1 shows the complete route for the preparation of the compound ІX. CHN analyses suggested a molecular formula of compound ІX (calc (%): C, 58.83; N, 14.70; H, 4.17), which reasonably agrees with the real amounts (%) of C (53.33), N (15.79), and H (4.34) (Scheme 1).

### 5.6. 2-(2-Aminophenyl)-1H-benzimidazole (III)

Yield: 97%; silver needle-shaped crystals; m.p: 203F02D204°C (213.5–214.0°C [56]). 1H NMR (DMSO-d6, *δ* ppm): 6.67 (1H, t, J = 8 Hz, Ar-H), 6.85 (1H, dd, J = 8.3 Hz, Ar-H), 7.14–7.18 (1H, t, J = 7.8 Hz Ar-H), 7.20–7.19 (1H, d,, J = 8 Hz, Ar-H), 7.21F02D7.22 (1H, d, J = 8 Hz, Ar-H),7.22–7.24 (1H, t,, J = 8.6 Hz, Ar-H), 7.32–7.24 (1H, t, J = 8 Hz, Ar-H), 7.59 (2H, s, NH2), 7.85–7.88 (1H, d, J = 8.0 Hz, Ar-H), 12.68 (1H, b, NH), and 13C NMR (DMSO-d6, *δ* ppm): 110.53, 111.23, 115.44, 116.58, 118.62, 121.86, 122.81, 127.72, 130.86, 134.01, 143.44, 148.71, and 153.01. IR (KBr, *νν*, cm−1): 3391 (NH, NH2), 2923 (CH aromatic), 1584 (C=N), 1448 (C=C), 783 (Ar-H), Anal. Calc for C13H11N3 (209.10 g/mol-1): C, H, N. (calc (%): N, 20.08; C, 74.62; H, 5.30), which reasonably agrees with the real amounts (%) of N (19.32), C (76.09), H (5.27).

### 5.7. 2-Chloro-3-Quinolinecarbaldehyde (VІ)

Yield: (94%); Yellowish solid; m.p.147-148°C. 1H NMR (DMSO-d6, *δ* ppm): 7.69 (1H, t, J = 8.2 Hz), 7.92 (1H, t, J = 8.5 Hz), 8.02 (1H, dd, J = 8.2 Hz), 8.11 (1H, d, J = 8.5 Hz), 8.80 (s, 1H), 10.60 (s, 1H), 13C NMR (DMSO-d6, *δ* ppm): 121.89, 122.35, 125.61, 129.37, 129.81, 131.16, 139.42, 150.71, 151.57, 192.63, Anal. Calc for C10H6ClNO (191.01 g/mol-1): C, H, N. (calc (%): N, 7.31; C, 62.68; H, 3.16), which reasonably agrees with the real amounts (%) of N (9.67), C (65.35), H (4.98).

### 5.8. (E)-N-[2-(1H-Benzimidazol-2-Yl) Phenyl]-1-(2-Chloro-3-Quinolinyl) Methanimine VII

Yield: (74%); Yellowish crystal. M.p.295–297°C. 1H NMR (DMSO-d6, *δ* ppm): 6.85–6.90 (1H, t, J = 7.8 Hz, CH), 6.97 (1H, d, J = 8.0 Hz, CH), 7.07 (1H, t, J = 7.6 Hz, CH), 7.19–7.33 (1H, t, J = 7.6 Hz, CH), 7.25–7.30 (1H, t, CH), 7.56 (1H, d, J = 2.1 Hz, CH), 7.66–7.63 (1H, d, J = 8.0 Hz, CH), 7.73–7.72 (1H, d, J = 8.0 Hz, CH), 7.89–7.83 (1H, t, J = 8.0 Hz, CH), 7.97–7.94 (1H, d, J = 8.0 Hz, CH), 7.99 (1H, s, -CH=N), 8.02–8.04(1H, d, J = 8.0 Hz, CH), 8.04–8.07(1H, d, J = 8.0 Hz, CH), 12.53 (1H, b, NH). 13C NMR (DMSO-d6, *δ* ppm): 66.62, 110.63, 111.86, 115.44, 118.92, 119.39, 122.85, 122.97, 125.21, 126.92, 128.02, 128.41, 128.93, 130.58, 132.28, 132.95, 137.64, 142.69, 147.35, 147.57, 148.36. IR (KBr, *νν*, cm−1): 3417 (NH), 3150 (CH aromatic), 1616 (C=N), 1499, 1589 (C=C), 1377 (C–N), 736 (C–Cl). MS m/z (%): 382 (20), 380 (40), 215 (70), 190 (98), 28 (100.00), Anal. Calc for C23H15ClN4 (382.10 g/mol-1): C, H, N. (calc (%): N, 14.63; C, 72.16; H, 3.95), which reasonably agrees with the real amounts (%) of N (14.89), C (70.14), H (3.24).

### 5.9. General Procedure for the Synthesis of the Chiral Benzimidazole Derivatives (Compound XI) with Trinuclear Cationic Copper (II) Complex (Compound ІX) as the Catalyst

First, the catalyst (1.5 mol%) was added to the round-bottom flask containing dry ethanol (green solvent) and was stirred for 15 min at room temperature. Then, L-amino acids and o-Phenylenediamine using a molar ratio of 1.4 : 1 were weighed and added to the reaction mixture and irradiated with microwave (green chemistry) at a power of 500 W for 20 min. After the completion of the reaction, it was monitored by TLC, and was removed as catalyst by filtration of the hot ethanol solution, and the products was recrystallized with ethanol. The physical and spectral data and the result of docking interaction Δ*G* (kcal/mol) of products are as follows:

#### 5.9.1. (1H-Benzo[d]imidazole-2-yl) Methanamine (a)

Yellowish solid, solid, yield 83%; m.p. 178–180°C (139.6–140.4°C [57]), Δ*G* = −6.98 kcal/mol, 1H NMR (DMSO-d6, *δ* ppm): 1.63 (2H, s, CH2), 4.56 (2H, s, NH2), 7.08–7.16 (2H, m, Ar-H), 7.45–7.50 (2H, t, Ar-H), 12.56 (1H, b, NH), 13C NMR (DMSO-d6, *δ* ppm): 45.36, 112.25, 113.52, 126.15, 140.35, 141.31, 164.88, Anal. Calc for C8H9N3 (147.08 g/mol-1): C, H, N. (calc (%): N, 28.55; C, 65.29; H, 6.16), which reasonably agrees with the real amounts (%) of N (27.66), C (66.32), H (6.02).

#### 5.9.2. (R)-1-(1H-Benzo[d]imidazole-2-yl)-2-Methylpropan-1-Amine (b)

Off-white powder. solid, yield 80%, m.p. 192–195°C (m.p. > 200°C [58]), Δ*G* = −7.35 kcal/mol, [*α*]24D = −35.0° (c 0.038, DMSO), 1HNMR (DMSO-d6, *δ* ppm): 0.78–0.79 (3H, d, J = 7 Hz, Ha in CH3), 0.79–0.80 (3H, d, J = 7 Hz, Hb in CH3), 1.54–1.55 (1H, d, CH), 4.10–4.20 (1H, m, CH), 2.22–2.27 (2H, m, NH2), 7.45–7.48 (2H, t, J = 6.0 Hz, Ar-H), 7.62–7.67(2H, m, Ar-H), 12.39 (1H, s, NH), 13C NMR (DMSO-d6, *δ* ppm): 20.10, 31.18, 63.43, 119.98, 123.55, 124.82, 138.72, 139.88, 164.58, Anal. Calc for C11H15N3 (189.13 g/mol-1): C, H, N. (calc (%): N, 22.20; C, 69.81; H, 7.99), which reasonably agrees with the real amounts (%) of N (23.14), C (70.53), H (6.33).

#### 5.9.3. (R)-1-(1H-Benzo[d]imidazol-2-yl)-3-Methylbutan-1-Amine (c)

White solid, yield 78%; m.p. 128–130°C, Δ*G* = −7.64 kcal/mol, [*α*]24D = −40.0° (c 0.035, DMSO), 1H NMR (DMSO-d6, *δ* ppm): 0.89–0.93 (6H, d, J = 5.1 Hz, 2CH3), 2.11–2.23 (1H, m, CH), 2.37–2.46 (2H, m, CH2), 3.38–3.46 (1H, q, CH), 5.51 (2H, s, NH2), 6.95–7.02 (2H, m, Ar-H), 7.35–7.45 (2H, m, Ar-H), 12.41 (1H, b, NH), 13C NMR (DMSO-d6, *δ* ppm): 22.70, 25.60, 46.70, 57.33, 117.08, 119.98, 124.15, 140.35, 141.62, 159.44, Anal. Calc for C12H17N3 (203.14 g/mol-1): C, H, N. (calc (%): N, 20.67; C, 70.90; H, 8.43), which reasonably agrees with the real amounts (%) of N (21.79), C (70.20), H (8.01).

#### 5.9.4. (R)-1-(1H-Benzo[d]imidazol-2-yl) Pentane-1,5-Diamine (d)

Yellowish solid, yield 66%; m.p. 188–190°C (m.p. > 200°C [59]), Δ*G* = −7.34 kcal/mol, [*α*]24D = −25.2° (c 0.032, DMSO), 1H NMR (DMSO-d6, *δ* ppm): 1.21–1.33 (2H, m, CH2), 1.88–2.01 (2H, m, CH2), 2.24–2.32 (2H, m, CH2), 2.83–2.86 (2H, m, CH2), 3.41–3.43 (1H, q, CH), 5.64 (2H, b, NH2), 7.41–7.44 (2H, m, Ar-H), 7.48–7.51 (2H, m, Ar-H), 12.52 (1H, b, NH), 13C NMR (DMSO-d6, *δ* ppm): 27.90, 32.73, 33.40, 42.40, 55.69, 117.08, 119.02, 124.15, 141.01, 141.62, 164.55, Anal. Calc for C12H18N4 (218.15 g/mol-1): C, H, N. (calc (%): N, 25.67; C, 66.02; H, 8.31), which reasonably agrees with the real amounts (%) of N (25.18), C (66.56), H (8.26).

#### 5.9.5. (R)-1-(1H-Benzo[d]imidazole-2-yl)-2-Phenylethanamine (e)

White solid, yield 87%.mp. 167–169°C (163.7–165.4°C [57]). Δ*G* = −7.68 kcal/mol, [*α*]24D = −35.8° (c 0.035, DMSO), 1H NMR (DMSO-d6, *δ* ppm): 0.74–0.87 (1H, q, CH), 1.16–1.26 (2H, m, CH2), 4.15 (2H, m, NH2), 7.28–7.33 (2H, m, Ar-H), 7.40–7.46 (2H, m, Ar-H), 7.60–7.70 (2H, m, Ar-H), 7.79-7.80 (1H, m, Ar-H), 12.48 (1H, s, NH), 13C NMR (DMSO-d6, *δ* ppm): 44.39, 54.20, 64.09, 115.45, 117.75, 121.89, 125.61, 129.37, 129.81, 139.42, 139.88, 157.45, Anal. Calc for C15H15N3 (237.13 g/mol-1): C, H, N. (calc (%): N, 17.71; C, 75.92; H, 6.37), which reasonably agrees with the real amounts (%) of N (19.20), C (76.95), H (4.85).

#### 5.9.6. (R)-2-Amino-2-(1H-Benzo[d]imidazole-2-yl) Ethanethiol (f)

White solid, yield 85% mp. 90–92°C. ΔG = −7.4 kcal/mol, [*α*]24D = −32.0° (c 0.029, DMSO), 1H NMR (DMSO-d6, *δ* ppm): 1.24 (1H, s, SH), 3.60–3.72 (1H, q, CH2), 3.73–3.76 (1H, q, CH2), 4.15–4.19 (1H, q, CH), 5.79 (2H, s, NH2), 7.45–7.54 (2H, m, Ar-H), 7.68–7.73 (2H, m, Ar-H), 12.52 (1H, s, NH), 13C NMR (DMSO-d6, *δ* ppm): 29.17, 61.80, 115.82, 117.39, 123.25, 135.15, 136.12, 163.61, Anal. Calc for C9H11N3S (193.07 g/mol-1): C, H, N. (calc (%): N, 21.74; C, 55.93; H, 5.74; S, 16.59), which reasonably agrees with the real amounts (%) of N (22.98), C (57.18), H (5.90).

#### 5.9.7. (R)-1-(1H-benzo[d]imidazol-2-yl)-3-(Methylthio) Propan-1-Amine (g)

Yellowish solid, yield 75% m.p. 71–73°C (71.9–73.7°C [57]), Δ*G* = −8.09 kcal/mol, [*α*]24D = −36.4° (c 0.030, DMSO), 1H NMR (DMSO-d6, *δ* ppm): 1.67 (3H, s, CH3), 1.77–1.86 (2H, m, CH2), 2.08–2.11 (2H, q, CH2), 3.87 (2H, b, NH2), 4.11–4.16 (1H, q, CH),7.43–7.48 (2H, m, Ar-H), 7.63–7.67 (2H, m, Ar-H), 12.23 (1H, s, NH), 13C NMR (DMSO-d6, *δ* ppm): 18.53, 31.18, 33.40, 57.33, 115.15, 118.35, 127.42, 138.72, 141.62, 161.01, Anal. Calc for C11H15N3S (221.10 g/mol-1): C, H, N. (calc (%): N, 18.99; C, 59.69; H, 6.83), which reasonably agrees with the real amounts (%) of N (19.90), C (61.65), H (7.32).

#### 5.9.8. (R)-Amino ((4-Amino-4-(1H-Benzo[d]imidazole-2-yl) Butyl) Amino) Methaniminium (h)

White solid, yield 80%. m.p. 155–158°C. Δ*G* = −7.39 kcal/mol, [*α*]24D = −48.5° (c 0.032, DMSO), 1H NMR (DMSO-d6, *δ* ppm): 1.84 (1H, b, NH), 1.97–2.08 (2H, m, CH2), 2.27–2.33 (2H, m, CH2), 2.51–2.60 (2H, t, CH2), 2.88–2.96 (1H, q, CH), 4.83 (2H, s, NH2), 7.42–7.46 (2H, t, Ar-H), 7.49–7.51 (2H, m, Ar-H), 10.01–10.18 (2H, m, NH2), 12.54 (1H, b, NH), 13C NMR (DMSO-d6, *δ* ppm): 23.07, 31.77, 55.03, 56.00, 112.85, 114.17, 119.98, 121.89, 136.48, 138.41, 141.01, 161.98, Anal. Calc for C12H19N6 (247.17 g/mol-1): C, H, N. (calc (%): N, 33.98; C, 58.28; H, 7.74), which reasonably agrees with the real amounts (%) of N (35.97), C (56.05), H (7.97).

#### 5.9.9. (R)-4-Amino-4-(1H-Benzo[d]imidazole-2-yl) Butanamide (k)

Brown solid, yield 75%. m.p. 169–171°C (m.p. > 200°C [59]), Δ*G* = −8.43 kcal/mol, [*α*]24D = −55.6° (c 0.035, DMSO), 1H NMR (DMSO-d6, *δ* ppm): 0.85–0.92 (1H, q, CH), 1.02–1.11 (2H, m, CH2), 4.22 (2H, s, NH2), 7.20–7.30 (2H, m, Ar-H), 7.44 (2H, s, NH2–C=O), 7.65–7.69 (2H, t, Ar-H), 12.65 (1H, b, NH), 13C NMR (DMSO-d6, *δ* ppm): 27.90, 32.73, 56.00, 114.17, 116.12, 122.88, 139.42, 140.35, 161.98, 176.54, Anal. Calc for C11H14N4O (218.12 g/mol-1): C, H, N. (calc (%): N, 25.67; C, 60.53; H, 6.47), which reasonably agrees with the real amounts (%) of N (28.09), C (62.18), H (4.73).

#### 5.9.10. (R)-3-Amino-3-(1H-Benzo[d]imidazole-2-yl) Propanamide (l)

Yellowish solid, yield 86% m.p. 157–159°C, Δ*G* = −10.99 kcal/mol, [*α*]24D = −43.0° (c 0.029, DMSO), 1H NMR (DMSO-d6, *δ* ppm): 0.82–0.94 (1H, q, CH), 1.08–1.50 (2H, q, CH2), 4.43 (2H, b, NH2), 7.20–7.32 (2H, m, Ar-H), 7.51–7.72 (2H, m, Ar-H), 7.55 (2H, s, NH2-C=O), 12.40 (1H, b, NH), 13C NMR (DMSO-d6, *δ* ppm): 43.43, 49.53, 115.45, 116.78, 125.61, 141.62, 143.91, 157.45, 171.34, Anal. Calc for C10H12N4O (204.10 g/mol-1): C, H, N. (calc (%): N, 27.43; C, 58.81; H, 5.92), which reasonably agrees with the real amounts (%) of N (28.64), C (60.79), H (4.57).

## 6. Conclusion

The current study attempted to design, synthesize, and investigate the biological activities of a series of novel Schiff base ligands (compound VII) of quinoline along with its Cu complex (compound VIII) and chiral benzimidazole derivatives as strong allosteric inhibitors of HIV-1, to obtain a drug enantiomer, which can reduce drug side effects and can have a greater therapeutic effect on this virus.

In addition, trinuclear cationic copper (II) complex (compound IX) was prepared as the catalyst by three strong arms to be exerted for the synthesis of chiral benzimidazole derivatives. This approach can offer excellent advantages such as the usage of nontoxic solvent and particularly reusable catalyst, as well as short reaction time, high yield, and eco-friendly chemistry approach when compared to other methods.

Considering the significant role of Mg^2+^ ion in the designing process, its coordination contributed to the recognition of potent HIV-1 integrase inhibitors.

The molecular docking studies revealed the interaction of HIV-1 integrase CCD dimer with the excellent binding affinities of synthesized compounds. The molecular dynamic simulation of compound VII and benzimidazole derivatives (compound XI) at the active site of protein implicated the factors of stability, inhibitors, and potential anti-HIV-1 agents. The stability of hydrogen-bond network, which was formed by the ligand in the active site of protein, increased the binding affinity and stabilization of the ligand with integrase.

## Figures and Tables

**Table 1:** Comparisons of trinuclear cationic copper (II) complex (compound IX) with the applied catalysts in the synthesis of benzimidazole derivatives.

Entry	Catalyst (mol %)	Solvent	Time	Temp. (°C)	Yield (%)	Ref
1	PPA and H_3_PO_4_	Solvent-free	24 h	180	75	[43]
2	HCl (6N)	Solvent-free	6 h	Reflux	66	[44]
3	Sncl_2_ (17.5)	Solvent-free	5 h	170	83	[45]
4	—	Toluene	9 h	85–95	97	[46]
5	HCl/H_2_O	Ethanol	9 h	85–95	68	[47]
6	PPA and H_3_PO_4_	Solvent-free	20 min	—	79	[48]
7	Compound IX	Ethanol	20 min	80	83	This work

Based on multicomponent reaction of glycine (1.4 mmol) and o-Phenylenediamine (1 mmol).

## Data Availability

The data used to support the findings of the study can be obtained from the journal site.
