# Supplementary material for: Discovery of New Ligand with Quinoline Scaffold as Potent Allosteric Inhibitor of HIV-1 and Its Copper Complexes as a Powerful Catalyst for the Synthesis of Chiral Benzimidazole Derivatives, and in Silico Anti-HIV-1 Studies
Source: Bioinorg Chem Appl. 2023 Apr 21;2023:2881582. doi: 10.1155/2023/2881582 (PMC10147532; doi:10.1155/2023/2881582)
Supplement: Supplementary Materials — Additional data can be found in this section. [file 2881582.f1.pdf]

Supporting Information

Discovery of new ligand with quinoline scaffold as potent allosteric inhibitor of HIV-1 and its copper complexes as a powerful catalyst for the synthesis of chiral benzimidazole derivatives, and *in silico* anti-HIV-1 studies.

Azimi G. Sabikeh [a], Ghodsieh Bagherzade [a]*, Mohammad Reza Saberi [b]*, Zeinab Amiri Tehranizadeh [b]

[a] *Department of Chemistry, Faculty of Sciences, University of Birjand, Birjand, 97175-615, Iran.*
[b] *Department of Medical Chemistry , School of Pharmacy, Mashhad University of Medical Sciences, 91775-1365, Mashhad, Iran.*

* Corresponding authors:
E-mail: bagherzadeh@birjand.ac.ir, Fax: +98 56 32345192; Tel: +98 56 32345192 .
E-mail: Saberimr@mums.ac.ir, Fax: +98-513-8823251; Tel: +98-513-8823255.

Spectra data

2-(2-Aminophenyl)-1H-benzimidazole (III),Yield: (97%); Silver needle-shaped crystals; m.p: 203-204°C(213.5-214.0°C). ^{1}H NMR (DMSO-d_6, δ ppm): 6.67 (1H, t, J = 8 Hz, Ar-H), 6.85 (1H, dd, J = 8.3 Hz, Ar-H), 7.14 – 7.18 (1H, t, J = 7.8 Hz Ar-H), 7.20 – 7.19 (1H, d, , J = 8 Hz, Ar-H), 7.21 – 7.22 (1H, d, , J = 8 Hz, Ar-H),7.22-7.24 (1H, t, , J = 8.6 Hz, Ar-H), 7.32-7.24 (1H, t, , J = 8 Hz, Ar-H), 7.59 (2H, s, NH_2), 7.85-7.88 (1H, d, J = 8.0 Hz, Ar-H) , 12.68 (1H, b, NH), ^{13}C NMR (DMSO-d6, δ ppm): 110.45, 111.51, 115.44, 116.49, 118.61, 121.77, 122.83, 127.89, 130.76, 134.15, 143.66, 148.95, 153.17. IR (KBr, ν, cm^{-1}): 3391 (NH, NH_2), 2923 (CH aromatic),1584 (C=N), 1448(C=C), 783 (Ar-H), Anal. Calc for $C_{13}H_{11}N_3$ (209.10 g/mol^{-1}): C, H, N. (calc (%): N, 20.08; C, 74.62; H, 5.30), which reasonably agrees with the real amounts (%) of N (19.32), C (76.09), H (5.27).

2-Chloro-3-quinolinecarbaldehyde (VI), Yield :(94%); Yellowish solid; m.p.147-148°C. ^{1}H NMR (DMSO-d_6, δ ppm): 7.69 (1H, t, J = 8.2 Hz), 7.92 (1H, t, J = 8.5 Hz), 8.02 (1H, dd, J = 8.2 Hz), 8.11 (1H, d, J = 8.5 Hz), 8.80 (s, 1H), 10.60 (s, 1H), ^{13}C NMR (DMSO-d6, δ ppm): 121.89, 122.35, 125.61, 129.37, 129.81, 131.16, 139.42, 150.57, 151.57, 192.63, Anal. Calc for $C_{10}H_6ClNO$ (191.01 g/mol^{-1}): C, H, N. (calc (%): N, 7.31; C, 62.68; H, 3.16), which reasonably agrees with the real amounts (%) of N (9.67), C (65.35), H (4.98).

(E)-N-[2-(1H-Benzimidazol-2-yl) phenyl]-1-(2-chloro-3- quinolinyl) methanimine VII, Yield:(74%); Yellowish crystal. m.p.295-297 °C. ^{1}H NMR (DMSO-d6, δ ppm): 6.85-6.90 (1H, t, J = 7.8 Hz, CH) , 6.97 (1H, d, J = 8.0 Hz, CH), 7.07 (1H, t, J = 7.6 Hz, CH), 7.19 –

7.33 (1H, t, J = 7.6 Hz, CH), 7.25-7.30(1H , t, CH), 7.56 (1H , d, J = 2.1 Hz, CH), 7.66-7.63 (1H, d, J = 8.0 Hz, CH), 7.73-7.72 (1H, d, J = 8.0 Hz, CH), 7.89 – 7.83 (1H , t, J = 8.0 Hz, CH), 7.97-7.94 (1H ,d , J = 8.0 Hz, CH), 7.99 (1H ,s, -CH=N), 8.02-8.04(1H , d, J = 8.0 Hz, CH), 8.04-8.07(1H ,d, J = 8.0 Hz, CH), 12.53 (1H, b, NH). ^{13}C NMR (DMSO-d_6, δ ppm): 66.57, 110.57, 111.89, 115.44, 118.87, 119.55, 122.83, 122.97, 125.32, 126.91, 128.11, 128.38, 128.91, 130.76, 131.55, 132.19, 132.98, 137.74, 142.72, 144.45, 147.47, 147.62, 148.42. IR (KBr, ν, cm^{-1}): 3417 (NH), 3150 (CH aromatic), 1616 (C=N), 1499, 1589 (C=C), 1377 (C-N), 736 (C-Cl). MS *m/z* (%): 382 (20), 380 (40), 215 (70), 190 (98), 28 (100.00), Anal. Calc for $C_{23}H_{15}ClN_4$ (382.10 g/mol^{-1}): C, H, N. (calc (%): N, 14,56; C, 71.78; H, 3.89), which reasonably agrees with the real amounts (%) of N (14.89), C (70.14), H (3.24).

(1H-benzo[d]imidazol-2-yl) methanamine (a), Yellowish solid, solid, yield 83 %; m.p. 178-180ºC (139.6-140.4ºC), ΔG= -6.98 kcal/mol, ^{1}H NMR (DMSO-d6, δ ppm): 1.63 (2H, s, CH2), 4.56 (2H, s, NH2), 7.08-7.16 (2H, m, Ar-H), 7.45-7.50 (2H, t, Ar-H), 12.56 (1H, b, NH), ^{13}C NMR (DMSO-d6, δ ppm): 45.36, 112.25, 113.52, 126.15, 140.35, 141.31, 164.88, Anal. Calc for $C_8H_9N_3$ (147.08 g/mol-1): C, H, N. (calc (%): N, 28.55; C, 65.29; H, 6.16), which reasonably agrees with the real amounts (%) of N (27.66), C (66.32), H (6.02).

(R)-1-(1H-benzo[d]imidazol-2-yl)-2-methylpropan-1-amine (b), Off-white powder. solid, yield 80 %, m.p. 192-195°C (m.p. > 200°C), ΔG= -7.35kcal/mol, [α]24D = -35.0° (c 0.038, DMSO), ^{1}H¬NMR (DMSO-d6, δ ppm): 0.78 – 0.79 (3H, d, J= 7 Hz, Ha in CH3), 0.79-0.80 (3H, d, J= 7 Hz, Hb in CH3), 1.54-1.55 (1H, d, CH), 4.10 – 4.20 (1H, m, CH), 2.22-2.27 (2H, m, NH2), 7.45-7.48 (2H, t, J = 6.0Hz, Ar-H), 7.62-7.67(2H, m, Ar-H), 12.39 (1H, s, NH), ^{13}C NMR (DMSO-d6, δ ppm): 20.10, 31.18, 63.43, 119.98, 123.55, 124.82, 138.72, 139.88, 164.58, Anal. Calc for $C_{11}H_{15}N_3$ (189.13 g/mol-1): C, H, N. (calc (%): N, 22.20; C, 69.81; H, 7.99), which reasonably agrees with the real amounts (%) of N (23.14), C (70.53), H (6.33).

(R)-1-(1H-benzo[d]imidazol-2-yl)-3-methylbutan-1-amine (c), White solid, yield 78 %; m.p. 128-130 ºC, ΔG= -7.64kcal/mol, [α]24D = -40.0° (c 0.035, DMSO), ^{1}H NMR (DMSO-d6, δ ppm): 0.89-0.93 (6H, d, J = 5.1 Hz, 2CH3), 2.11-2.23 (1H, m, CH), 2.37-2.46 (2H, m, CH2), 3.38-3.46 (1H, q, CH), 5.51 (2H, s, NH2), 6.95-7.02 (2H, m, Ar-H), 7.35-7.45 (2H, m, Ar-H), 12.41 (1H, b, NH), ^{13}C NMR (DMSO-d6, δ ppm): 22.70, 25.60, 46.70, 57.33, 117.08, 119.98, 124.15, 140.35, 141.62, 159.44, Anal. Calc for $C_{12}H_{17}N_3$ (203.14 g/mol-1): C, H, N. (calc (%): N, 20.67; C, 70.90; H, 8.43), which reasonably agrees with the real amounts (%) of N (21.79), C (70.20), H (8.01).

(R)-1-(1H-benzo[d]imidazol-2-yl) pentane-1,5-diamine (d), Yellowish solid, yield 66 %; m.p. 188-190 ºC (m.p. > 200°C), ΔG= -7.34kcal/mol, [α]24D = -25.2° (c 0.032, DMSO), ^{1}H NMR (DMSO-d6, δ ppm): 1.21-1.33 (2H, m, CH2), 1.88-2.01 (2H, m, CH2), 2.24-2.32 (2H, m, CH2), 2.83-2.86 (2H, m, CH2), 3.41-3.43 (1H, q, CH), 5.64 (2H, b, NH2), 7.41-7.44 (2H, m, Ar-H), 7.48-7.51 (2H, m, Ar-H), 12.52 (1H, b, NH), ^{13}C NMR (DMSO-d6, δ ppm): 27.90, 32.73, 33.40, 42.40, 55.69, 117.08, 119.02, 124.15, 141.01, 141.62, 164.55, Anal. Calc for $C_{12}H_{18}N_4$ (218.15 g/mol-1): C, H, N. (calc (%): N, 25.67; C, 66.02; H, 8.31), which reasonably agrees with the real amounts (%) of N (25.18), C (66.56), H (8.26).

(R)-1-(1H-benzo[d]imidazol-2-yl)-2-phenylethanamine (e), White solid, yield 87 % .mp. 167-169 ºC (163.7-165.4ºC). ΔG= -7.68 kcal/mol, [α]24D = -35.8° (c 0.035, DMSO), ^{1}H NMR (DMSO-d6, δ ppm): 0.74-0.87 (1H, q, CH), 1.16-1.26 (2H, m, CH2), 4.15 (2H, m, NH2), 7.28-7.33 (2H, m, Ar-H), 7.40-7.46 (2H, m, Ar-H), 7.60-7.70 (2H, m, Ar-H), 7.79-7.80 (1H, m, Ar-H), 12.48 (1H, s, NH), ^{13}C NMR (DMSO-d6, δ ppm): 44.39, 54.20, 64.09, 115.45, 117.75, 121.89, 125.61, 129.37, 129.81, 139.42, 139.88, 157.45, Anal. Calc for $C_{15}H_{15}N_3$ (237.13 g/mol-1): C, H, N. (calc (%): N, 17.71; C, 75.92; H, 6.37), which reasonably agrees with the real amounts (%) of N (19.20), C (76.95), H (4.85).

(R)-2-amino-2-(1H-benzo[d]imidazol-2-yl) ethanethiol (f), white solid, yield 85 % .mp. 90-92 ºC. ΔG= -7.4 kcal/mol, [α]24D = -32.0° (c 0.029, DMSO), ^{1}H NMR (DMSO-d6, δ ppm): 1.24 (1H, s, SH), 3.60-3.72 (1H, q, CH2), 3.73-3.76 (1H, q, CH2), 4.15-4.19 (1H, q, CH), 5.79 (2H, s, NH2), 7.45-7.54 (2H, m, Ar-H), 7.68-7.73 (2H, m, Ar-H), 12.52 (1H, s, NH), ^{13}C NMR (DMSO-d6, δ ppm): 29.17, 61.80, 115.82, 117.39, 123.25, 135.15, 136.12, 163.61, Anal. Calc for $C_9H_{11}N_3S$ (193.07 g/mol-1): C, H, N. (calc (%): N, 21.74; C, 55.93; H, 5.74; S, 16.59), which reasonably agrees with the real amounts (%) of N (22.98), C (57.18), H (5.90).

(R)-1-(1H-benzo[d]imidazol-2-yl)-3-(methylthio) propan-1-amine (g), Yellowish solid, yield 75 % m.p. 71-73 ºC (71.9-73.7 ºC), ΔG= -8.09 kcal/mol, [α]24D = -36.4° (c 0.030, DMSO), ^{1}H NMR (DMSO-d6, δ ppm): 1.67 (3H, s, CH3), 1.77-1.86 (2H, m, CH2), 2.08-2.11 (2H, q, CH2), 3.87 (2H, b, NH2), 4.11-4.16 (1H, q, CH),7.43-7.48 (2H, m, Ar-H), 7.63-7.67 (2H, m, Ar-H), 12.23 (1H, s, NH), ^{13}C NMR (DMSO-d6, δ ppm): 18.53, 31.18, 33.40, 57.33, 115.15, 118.35, 127.42, 138.72, 141.62, 161.01, Anal. Calc for $C_{11}H_{15}N_3S$ (221.10 g/mol-1): C, H, N. (calc (%): N, 18.99; C, 59.69; H, 6.83), which reasonably agrees with the real amounts (%) of N (19.90), C (61.65), H (7.32).

(R)-amino((4-amino-4-(1H-benzo[d]imidazol-2-yl) butyl) amino) methaniminium (h), White solid, yield 80 %. m.p. 155-158ºC. ΔG= -7.39 kcal/mol, [α]24D = -48.5° (c 0.032, DMSO), ^{1}H NMR (DMSO-d6, δ ppm): 1.84 (1H, b, NH), 1.97-2.08 (2H, m, CH2), 2.27-2.33 (2H, m, CH2), 2.51-2.60 (2H, t, CH2), 2.88-2.96 (1H, q, CH), 4.83 (2H, s, NH2), 7.42-7.46 (2H, t, Ar-H), 7.49-7.51 (2H, m, Ar-H), 10.01-10.18 (2H, m, NH2), 12.54 (1H, b, NH), ^{13}C NMR (DMSO-d6, δ ppm): 23.07, 31.77, 55.03, 56.00, 112.85, 114.17, 119.98, 121.89, 136.48, 138.41, 141.01, 161.98, Anal. Calc for $C_{12}H_{1}9N_6$ (247.17 g/mol-1): C, H, N. (calc (%): N, 33.98; C, 58.28; H, 7.74), which reasonably agrees with the real amounts (%) of N (35.97), C (56.05), H (7.97).

(R)-4-amino-4-(1H-benzo[d]imidazol-2-yl) butanamide(k), Brown solid, yield 75%. m.p. 169-171ºC (m.p. > 200°C), ΔG= -8.43kcal/mol, [α]24D = -55.6° (c 0.035, DMSO), ^{1}H NMR (DMSO-d6, δ ppm): 0.85-0.92 (1H, q, CH), 1.02-1.11 (2H, m, CH2), 4.22 (2H, s, NH2), 7.20-7.30 (2H, m, Ar-H), 7.44 (2H, s, NH2–C=O), 7.65-7.69 (2H, t, Ar-H), 12.65 (1H, b, NH), ^{13}C NMR (DMSO-d6, δ ppm): 27.90, 32.73, 56.00, 114.17, 116.12, 122.88, 139.42, 140.35, 161.98, 176.54, Anal. Calc for $C_{11}H_{14}N_4O$ (218.12 g/mol-1): C, H, N. (calc (%): N, 25.67; C, 60.53; H, 6.47), which reasonably agrees with the real amounts (%) of N (28.09), C (62.18), H (4.73).

(R)-3-amino-3-(1H-benzo[d]imidazol-2-yl) propanamide (l). Yellowish solid, yield 86 % m.p. 157-159 ºC, ΔG= -10.99 kcal/mol, [α]24D = -43.0° (c 0.029, DMSO), ^{1}H NMR (DMSO-d6, δ ppm): 0.82-0.94 (1H, q, CH), 1.08-1.50 (2H, q , CH2), 4.43 (2H, b, NH2), 7.20-7.32 (2H, m, Ar-H), 7.51-7.72 (2H, m, Ar-H), 7.55 (2H, s, NH2–C=O), 12.40 (1H, b, NH), ^{13}C NMR (DMSO-d6, δ ppm): 43.43, 49.53, 115.45, 116.78, 125.61, 141.62, 143.91, 157.45, 171.34, Anal. Calc for $C_{10}H_{12}N_4O$ (204.10 g/mol-1): C, H, N. (calc (%): N, 27.43; C, 58.81; H, 5.92), which reasonably agrees with the real amounts (%) of N (28.64), C (60.79), H (4.57).

Green chemistry metrics analysis

The following formulae were used for calculating atom economy (AE), atom efficiency (AEf), carbon efficiency (CE), reaction mass efficiency (RME), optimum efficiency (OE), process mass intensity (PMI), E factor, solvent and water intensity (SI and WI).

$$AE = \frac{\text{Molecular weight of the product}}{\text{Total molecular weight of reactants}} \times 100$$

$$AE_f = AE \cdot Yield\ (\%)$$

$$CE = \frac{\text{Amount of carbon in the product}}{\text{Total carbon present in reactants}}$$

$$RME = \frac{\text{Mass of isolated product}}{\text{Total mass of reactants}} \times 100$$

$$OE = \frac{\text{RME}}{\text{AE}} \times 100$$

$$PMI = \frac{\text{Total mass of input material in the whole process}}{\text{Mass of product}}$$

$$E\ factor = PMI - 1$$

$$SI = \frac{\text{Total mass of solvents excluding water in the whole process}}{\text{Mass of product}}$$

$$WI = \frac{\text{Total mass of water used in the whole process}}{\text{Mass of product}}$$

Synthesis of 2-(2-Aminophenyl)-1H-benzimidazole:

Experimental procedure:

3 mmol *o*-phenylenediamine I and 3 mmol anthranilic acid II and 5g polyphosphoric acid (PPA) were added and mixed with a mechanical mixer until was obtained a uniform paste. The reaction mixture was reacted with the microwave-assisted and solvent free (green chemistry) with an output power of 1000 W for 12 min at 160 °C.

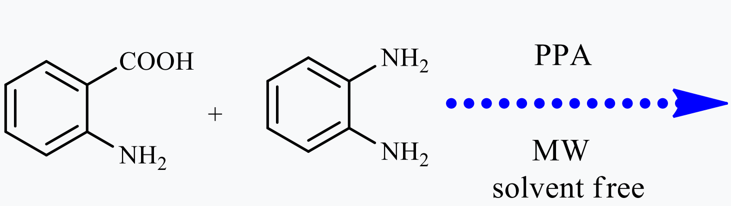

Chemical Formula:	Chemical Formula:	Chemical Formula:
$C_7H_7NO_2$	$C_6H_8N_2$	$C_{13}H_{11}N_3$
Molecoular Weight:	Molecoular Weight:	Molecoular Weight:
137.05	108.07	209.10

Materials used for metrics calculations:

anthranilic acid (0.137 g, 1 mmol), Phenylenediamine (0.108 g, 1mmol), solvent free, product (0.202g, 0.97 mmol).

$$AE = \frac{209.10}{137.05 + 108.07} \times 100 = 85.30$$

$$AEf = 85.30 \times 97\% = 82.74$$

$$CE = \frac{13 \times 0.00097}{7 \times 0.001 + 6 \times 0.001} \times 100 = 97$$

$$RME = \frac{0.202}{0.137 + 0.108} \times 100 = 82.44$$

$$OE = \frac{82.44}{85.30} \times 100 = 96.64$$

$$PMI = \frac{0.137 + 0.108}{0.202} = 1.21$$

$$E\ factor = 1.21 - 1 = 0.21$$

$$SI = 0$$

$$WI = 0$$

Synthesis of (E)-N-[2-(1H-Benzimidazol-2-yl) phenyl]-1-(2-chloro-3-quinolinyl) methanimine:

Experimental procedure:

The desired Schiff base ligand was performed with high efficiency by reaction (5mmol) of the 2-Chloro-3-quinolinecarbaldehyde VI and (5mmol) of the 2-(2-Aminophenyl)-1H-benzimidazole III at room temperature in the presence (20µl) of the 1,8- diazabicyclo (5.4.0) undec-7-ene (DBU) in dry ethanol is cheap and available green solvent**.**

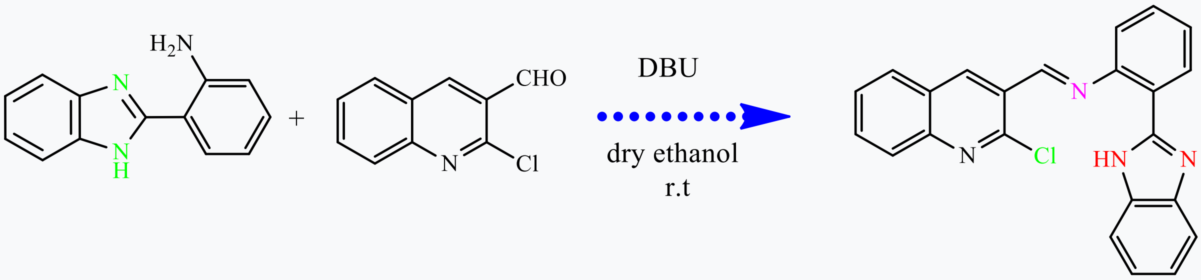

Chemical Formula:	Chemical Formula:	Chemical Formula:
$C_{13}H_{11}N_3$	$C_{10}H_6ClNO$	$C_{23}H_{15}ClN_4$
Molecoular Weight:	Molecoular Weight:	Molecoular Weight:
209.10	191.01	382.10

Materials used for metrics calculations:

2-(2-Aminophenyl)-1H-benzimidazole (0.209 g, 1 mmol), 2-Chloro-3-quinolinecarbaldehyde (0.191 g, 1mmol), dry ethanol (1.576g, 2ml), product (0.282g, 0.74 mmol).

$$AE = \frac{382.10}{209.10 + 191.01} \times 100 = 95.49$$

$$AE_f = 95.49 \times 74\% = 70.66$$

$$CE = \frac{23 \times 0.00074}{13 \times 0.001 + 10 \times 0.001} \times 100 = 74$$

$$RME = \frac{0.282}{0.209 + 0.191} \times 100 = 70.5$$

$$OE = \frac{70.5}{95.49} \times 100 = 73.82$$

$$PMI = \frac{0.209 + 0.191 + 1.576}{0.282} = 7$$

$$E\ factor = 7 - 1 = 6$$

$$SI = \frac{2 \times 0.788}{0.282} = 5.58$$

$$WI = 0$$

General procedure for the synthesis of the chiral benzimidazole derivatives (compound XI) with Trinuclear cationic copper(II) complex (compound IX) as the catalyst:

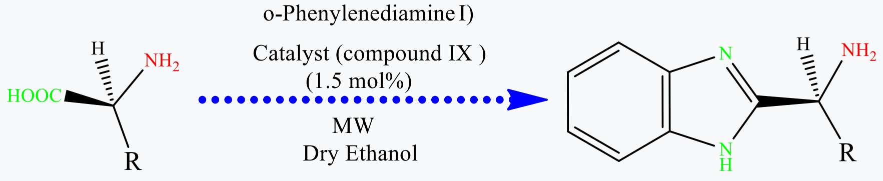

(X) (XI)

Experimental procedure:

First, the catalyst (1.5 mol%) was added to the round-bottom flask containing dry ethanol (green solvent) and was stirred for 15 min at room temperature. Then, L-amino acids and o-Phenylenediamine using the molar ratio of 1.4: 1 was weighed and added to the reaction mixture and irradiated with microwave (green chemistry) at a power of 500 W for 20 min. After the completion of the reaction, was monitored by TLC, and then was removed catalyst by filtration of the hot ethanol solution, and the products was recrystallized with ethanol.

Synthesis of (R)-3-amino-3-(1H-benzo[d]imidazol-2-yl) propanamide:

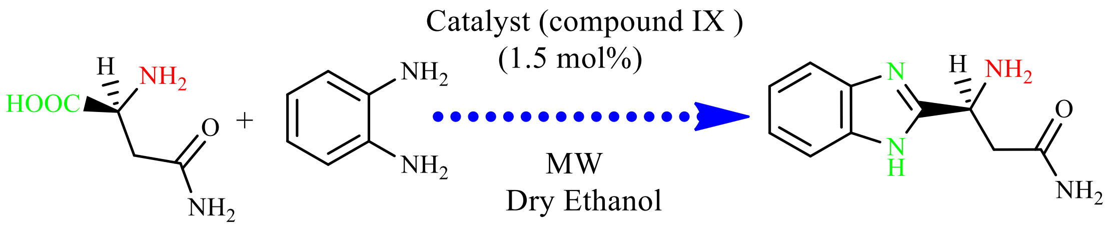

Chemical Formula:	Chemical Formula:	Chemical Formula:
$C_4H_8N_2O_3$	$C_6H_8N_2$	$C_{10}H_{12}N_4O$
Molecoular Weight:	Molecoular Weight:	Molecoular Weight:
132.05	108.07	204.10

Materials used for metrics calculations:

Asparagine (0.184 g, 1.4 mmol), Phenylenediamine (0.108 g, 1mmol), dry ethanol (1.576g, 2ml), product (0.175g, 0.86 mmol).

$$AE = \frac{204.10}{184.87 + 108.07} \times 100 = 69.67$$

$$AE_f = 69.67 \times 86\% = 59.91$$

$$CE = \frac{10\ \times\ 0.00086}{4 \times\ 0.0014 + 6 \times 0.001} \times 100 = 74$$

$$RME = \frac{0.175}{0.184 + 0.108} \times 100 = 60$$

$$OE = \frac{60}{69.67} \times 100 = 86$$

$$PMI = \frac{0.184 + 0.108 + 1.576}{0.175} = 10.67$$

$E\ factor$ = 10.67 – 1= 9.67

$$SI = \frac{2 \times 0.788}{0.175} = 9$$

$$WI = 0$$

Synthesis of (R)-2-amino-2-(1H-benzo[d]imidazol-2-yl) ethanethiol:

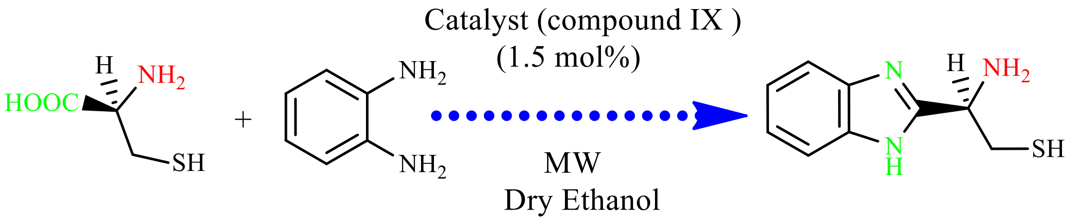

Chemical Formula:	Chemical Formula:	Chemical Formula:
$C_3H_7NO_2S$	$C_6H_8N_2$	$C_9H_{11}N_3S$
Molecoular Weight:	Molecoular Weight:	Molecoular Weight:
121.02	108.07	193.07

Materials used for metrics calculations:

cysteine (0.169 g, 1.4 mmol), Phenylenediamine (0.108 g, 1mmol), dry ethanol (1.576g, 2ml), product (0.164g, 0.85 mmol).

$$AE = \frac{193.07}{108.07 + 169.42} \times 100 = 84.86$$

$AE_f = 84.86 \times 85\% = 72.13$

$$CE = \frac{9 \times 0.00085}{3 \times 0.0014 + 6 \times 0.001} = 75$$

$$RME = \frac{0.164}{0.169 + 0.108} \times 100 = 72.24$$

$$OE = \frac{72.24}{84.86} \times 100 = 85$$

$$PMI = \frac{0.169 + 0.108 + 1.576}{0.164} = 11.29$$

$E\ factor$ = 11.29 – 1= 10.29

$$SI = \frac{2 \times 0.788}{0.164} = 9.6$$

$$WI = 0$$

Synthesis of (R)-amino((4-amino-4-(1H-benzo[d]imidazol-2-yl) butyl) amino) methaniminium:

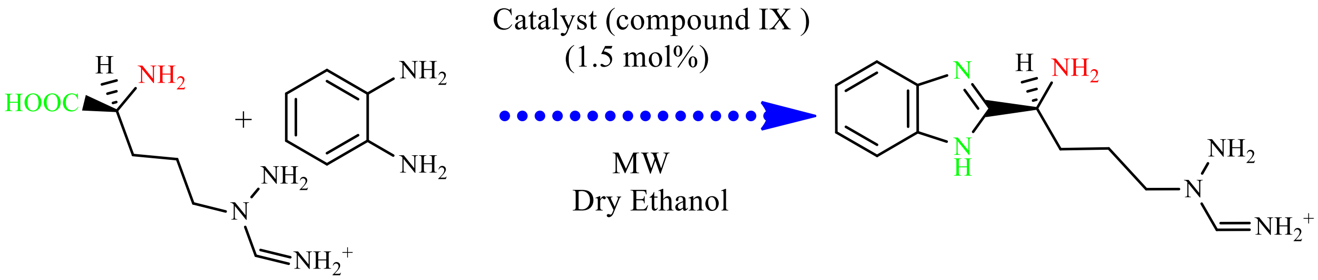

Chemical Formula:	Chemical Formula:	Chemical Formula:
$C_6H_{15}N_4O_2^+$	$C_6H_8N_2$	$C_{12}H_{19}N_6^+$
Molecoular Weight:	Molecoular Weight:	Molecoular Weight:
175.12	108.07	247.17

Materials used for metrics calculations:

Arginine (0.245 g, 1.4 mmol), Phenylenediamine (0.108 g, 1mmol), dry ethanol (1.576g, 2ml), product (0.197g, 0.80 mmol).

$$AE = \frac{247.17}{245.16 + 108.07} \times 100 = 69.97$$

$AE_f = 69.97 \times\ 80\% = 55.97$

$$CE = \frac{12\ \times 0.00080}{6 \times 0.0014 + 6\ \times 0.001} \times 100 = 66.66$$

$$RME = \frac{0.197}{0.245 + 0.108} \times 100 = 55.80$$

$$OE = \frac{55.80}{69.97} \times 100 = 79.74$$

$$PMI = \frac{0.245 + 0.108 + 1.576}{0.197} = 9.7$$

$E\ factor = 9.7 - 1 = 8.7$

$$SI = \frac{2\ \times 0.788}{0.197} = 8$$

$WI = 0$

Synthesis of (1H-benzo[d]imidazol-2-yl) methanamine:

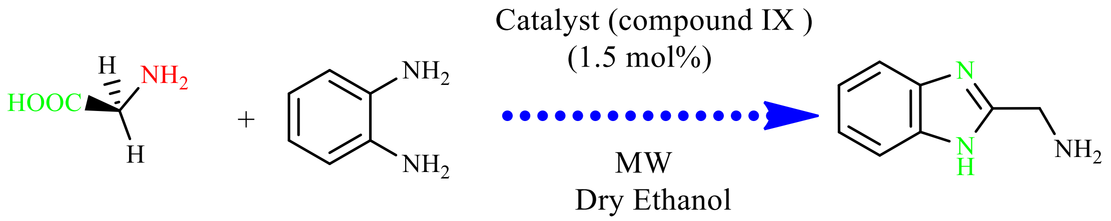

Chemical Formula:	Chemical Formula:	Chemical Formula:
$C_2H_5NO_2$	$C_6H_8N_2$	$C_8H_9N_3$
Molecoular Weight:	Molecoular Weight:	Molecoular Weight:
75.03	108.07	147.08

Materials used for metrics calculations:

Glycine (0.105 g, 1.4 mmol), Phenylenediamine (0.108 g, 1mmol), dry ethanol (1.576g, 2ml), product (0.122g, 0.83 mmol).

$$AE = \frac{147.08}{105.04 + 108.07} \times 100 = 69.01$$

$AE_f = 69.01 \times 83\% = 57.28$

$$CE = \frac{8 \times 0.00083}{2 \times 0.0014 + 6 \times 0.001} \times 100 = 75.45$$

$$RME = \frac{0.122}{0.105 + 0.108} \times 100 = 47.28$$

$$OE = \frac{47.28}{69.01} \times 100 = 68.51$$

$$PMI = \frac{0.105 + 0.108 + 1.576}{0.122} = 14.66$$

E factor = 14.66– 1= 13.66

$$SI = \frac{2 \times 0.788}{0.122} = 12.91$$

$$WI = 0$$

Synthesis of (R)-1-(1H-benzo[d]imidazol-2-yl)-2-phenylethanamine:

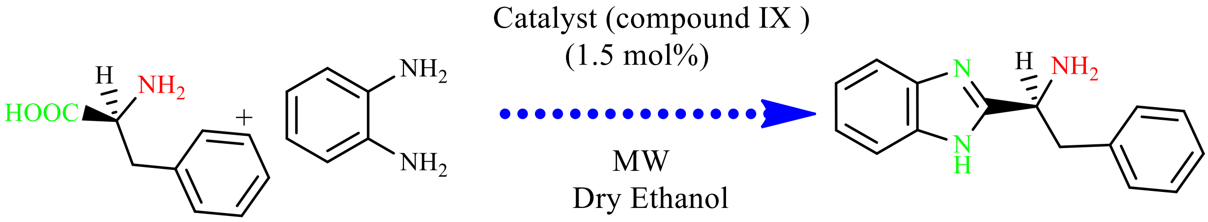

Chemical Formula:	Chemical Formula:	Chemical Formula:
$C_9H_{11}NO_2$	$C_6H_8N_2$	$C_{15}H_{15}N_3$
Molecoular Weight:	Molecoular Weight:	Molecoular Weight:
165.08	108.07	237.13

Materials used for metrics calculations:

Phenylalanine (0.231 g, 1.4 mmol), Phenylenediamine (0.108 g, 1mmol), dry ethanol (1.576g, 2ml), product (0.206g, 0.87 mmol).

$$AE = \frac{237.13}{231.11 + 108.07} \times 100 = 69.91$$

AE_f = 69.91 × 87%= 60.82

$$CE = \frac{15 \times 0.00087}{9 \times 0.0014 + 6 \times 0.001} \times 100 = 69.89$$

$$RME = \frac{0.206}{0.231 + 0.108} \times 100 = 60.76$$

$$OE = \frac{60.76}{69.91} \times 100 = 86.91$$

$$PMI = \frac{0.231 + 0.108 + 1.576}{0.206} = 9.29$$

E factor = 9.29– 1= 8.29

$$SI = \frac{2 \times 0.788}{0.206} = 7.65$$

$$WI = 0$$

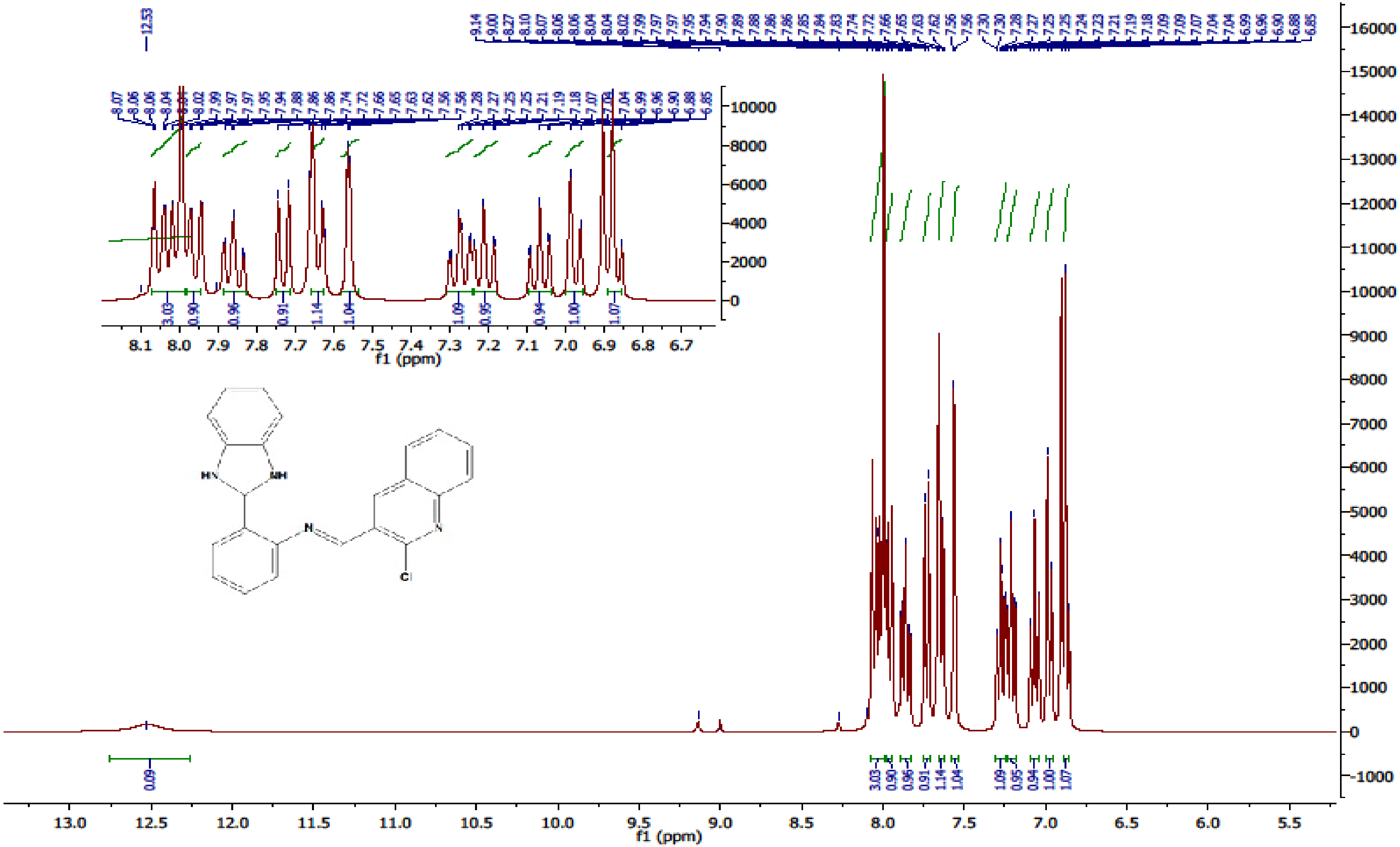

Figure S1: ^{1}H-NMR of (E)-N-[2-(1H-Benzimidazol-2-yl) phenyl]-1-(2-chloro-3- quinolinyl) methanimine.

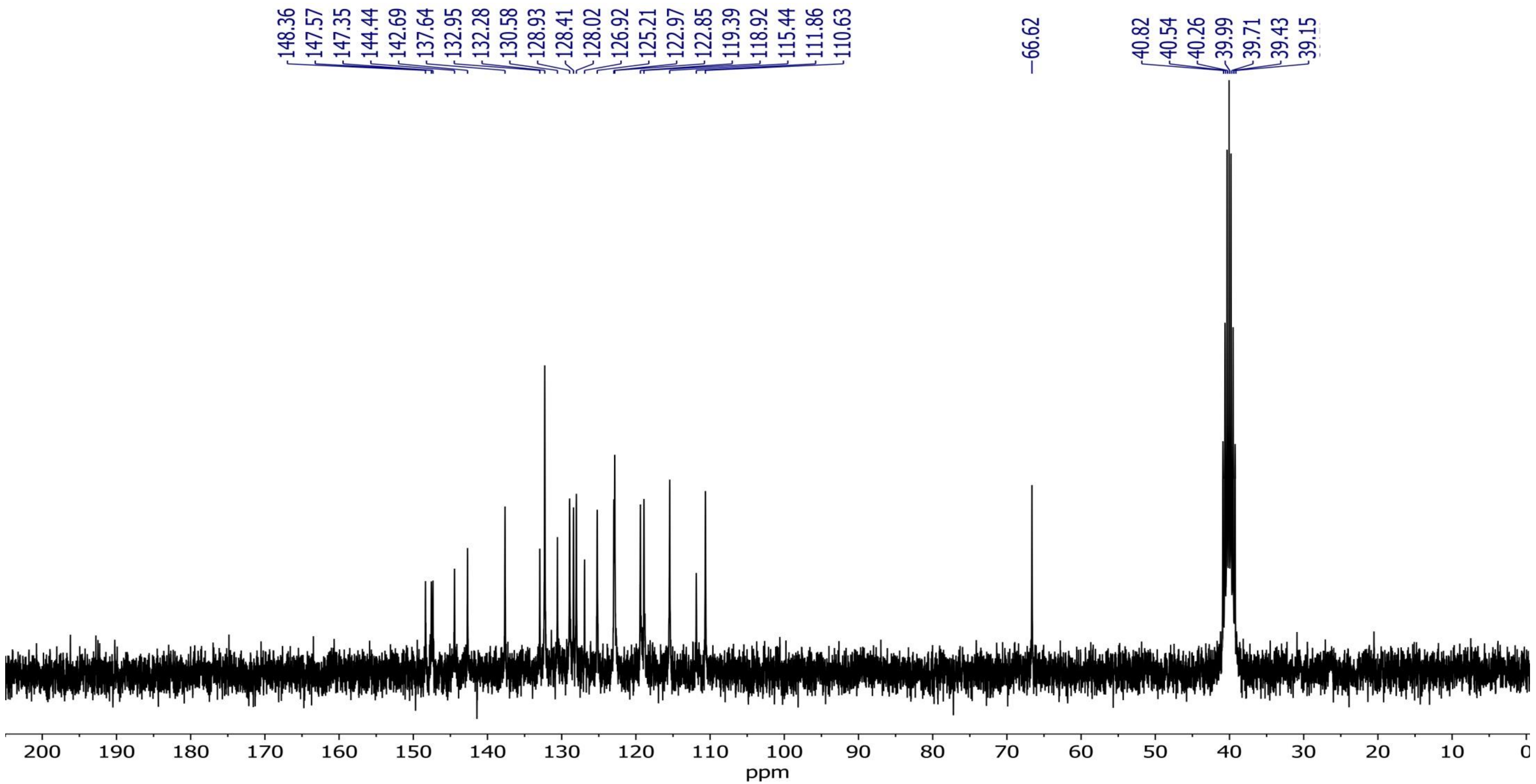

Figure S2: ^{13}C-NMR of (E)-N-[2-(1H-Benzimidazol-2-yl) phenyl]-1-(2-chloro-3- quinolinyl) methanimine.

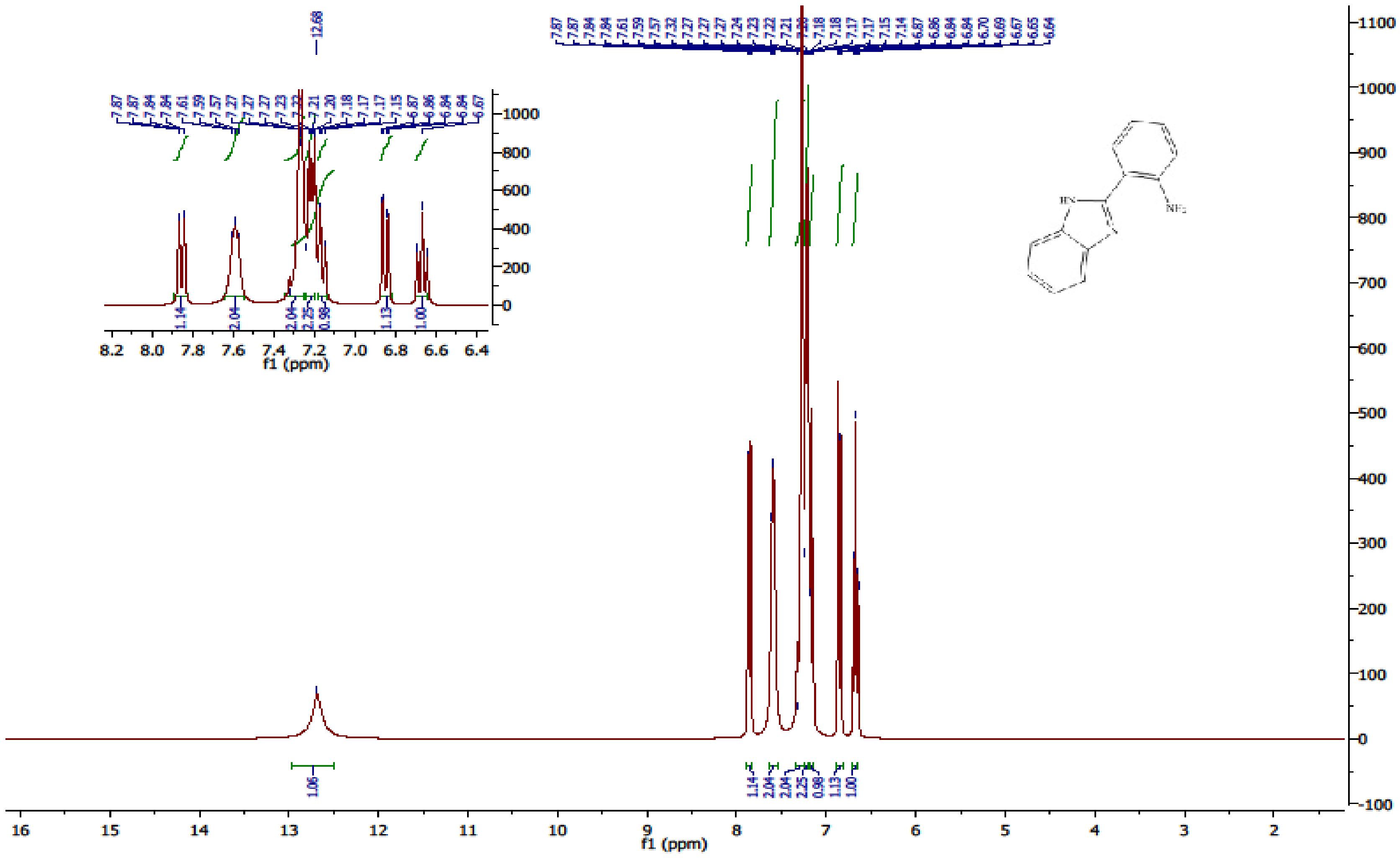

Figure S3: ^{1}H-NMR of 2-(2-Aminophenyl)-1H-benzimidazole

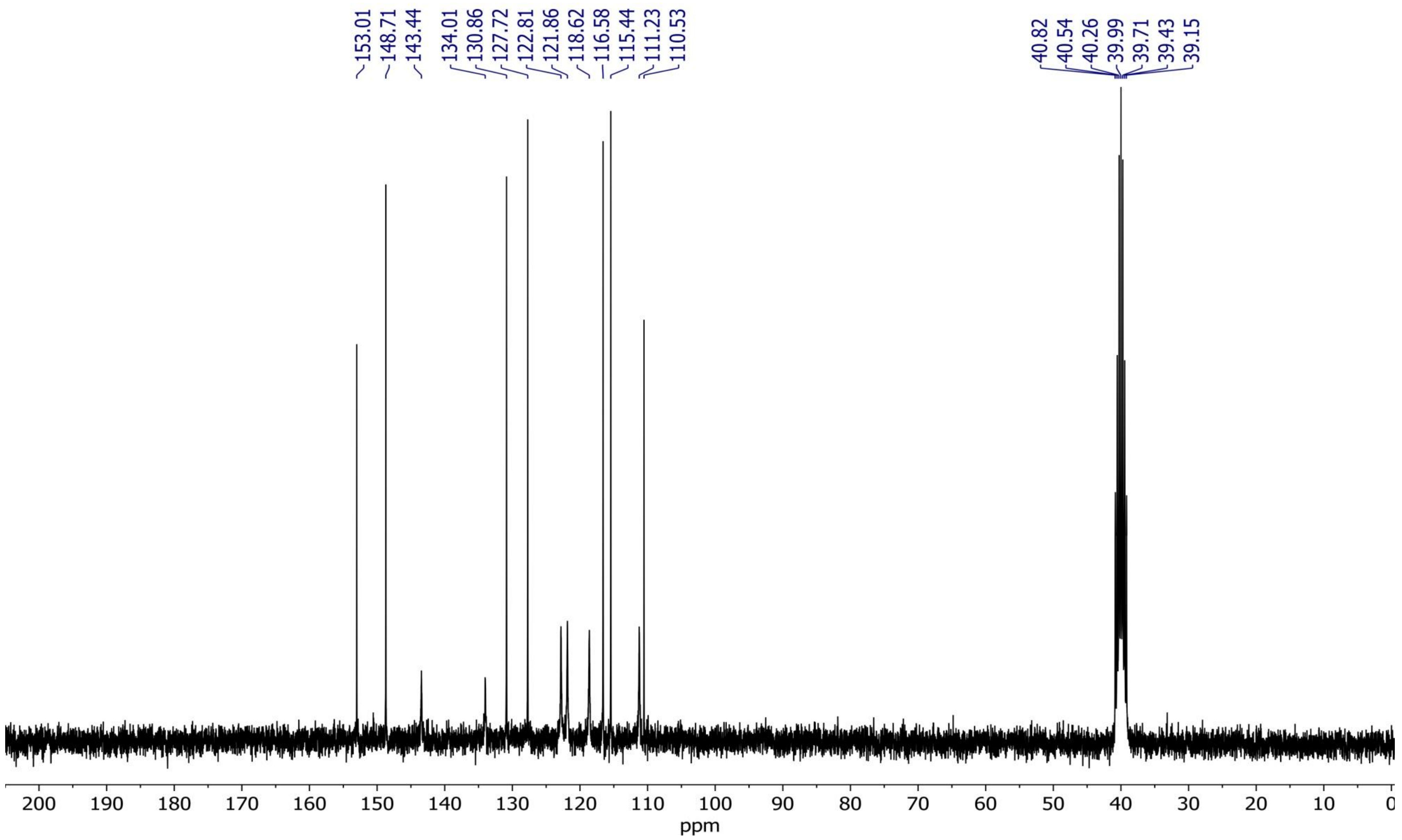

Figure S4: ^{13}C-NMR of 2-(2-Aminophenyl)-1H-benzimidazole

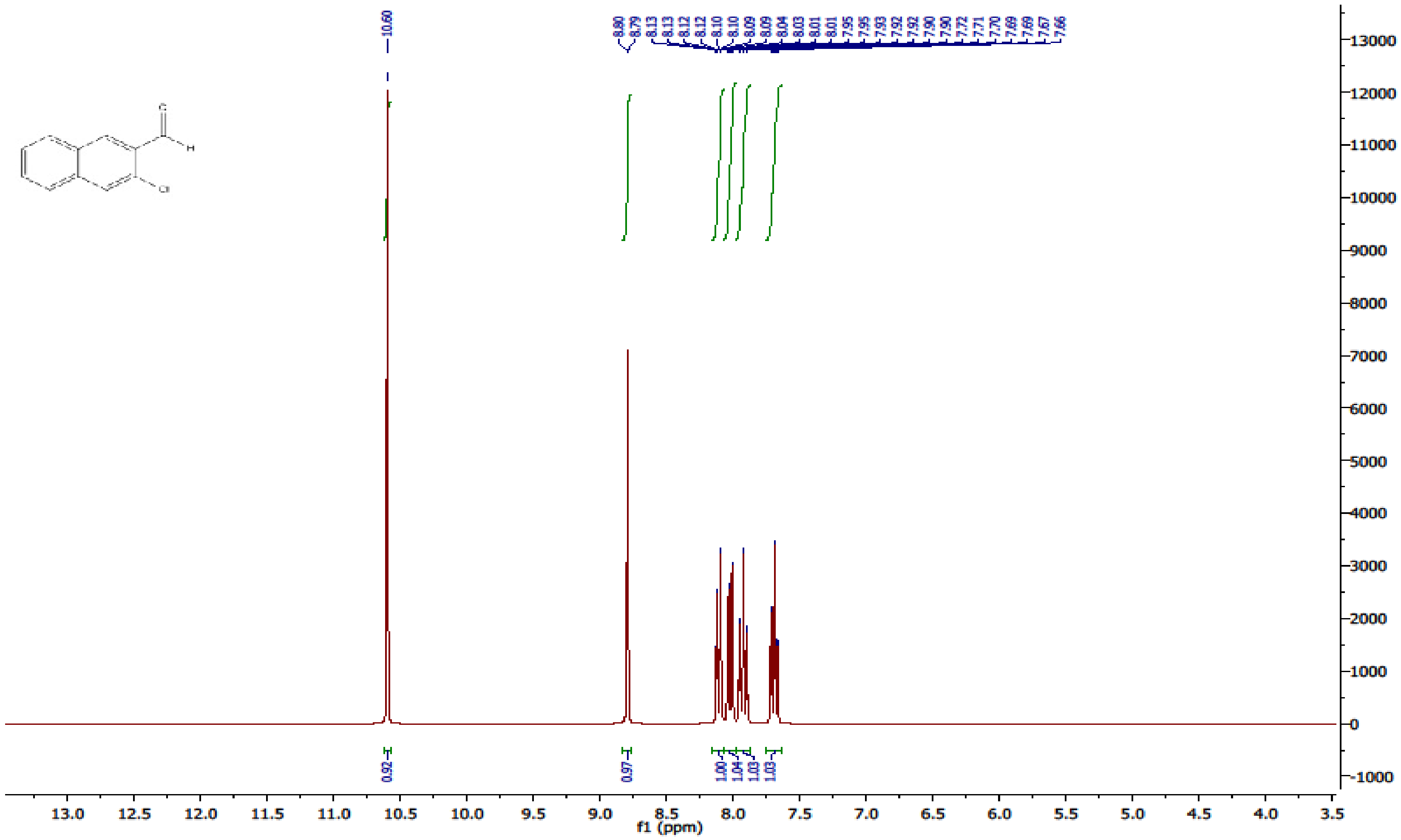

Figure S5: ¹H-NMR of 2-Chloro-3-quinolinecarbaldehyde.

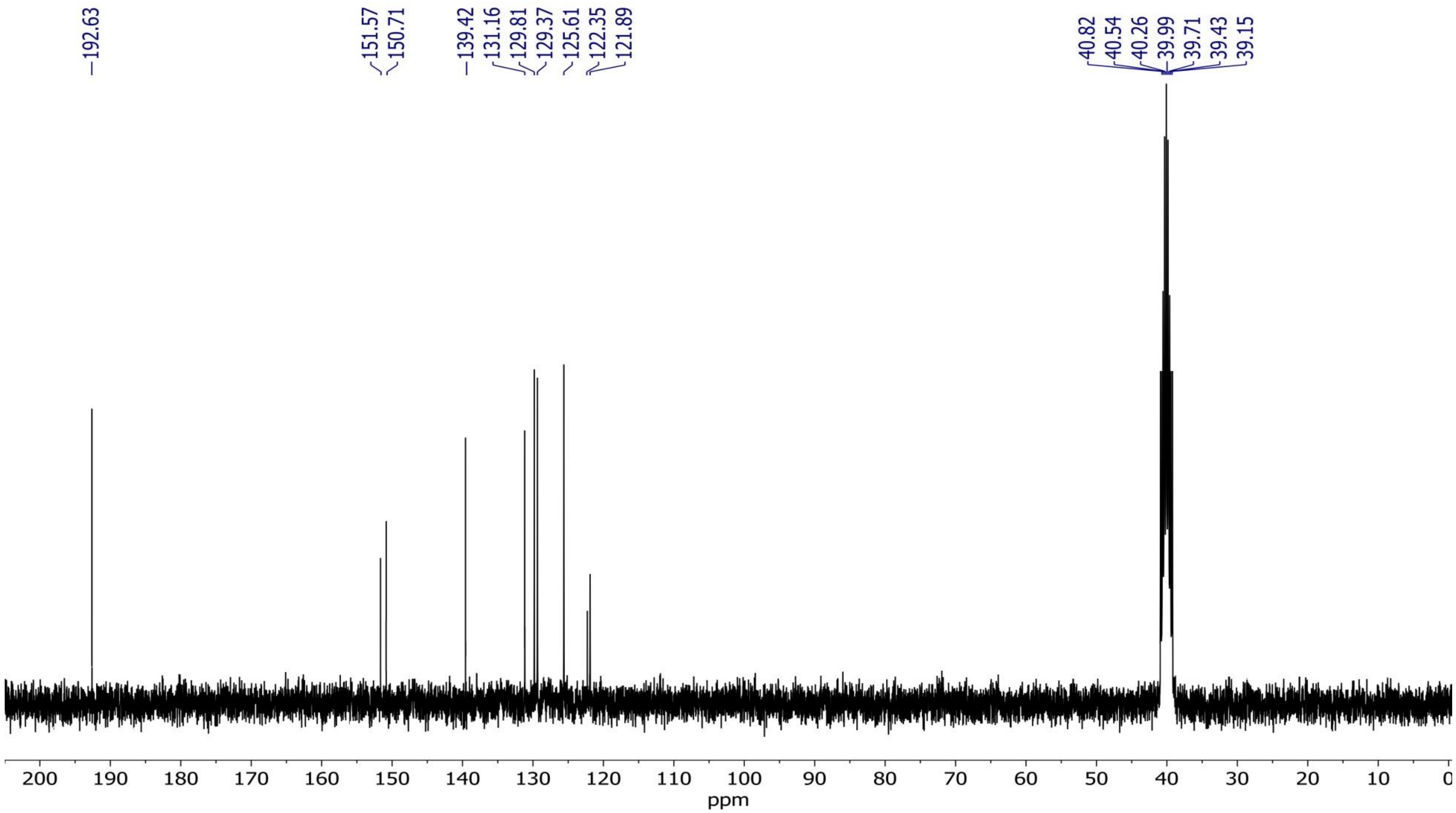

Figure S6: ^{13}C-NMR of 2-Chloro-3-quinolinecarbaldehyde.

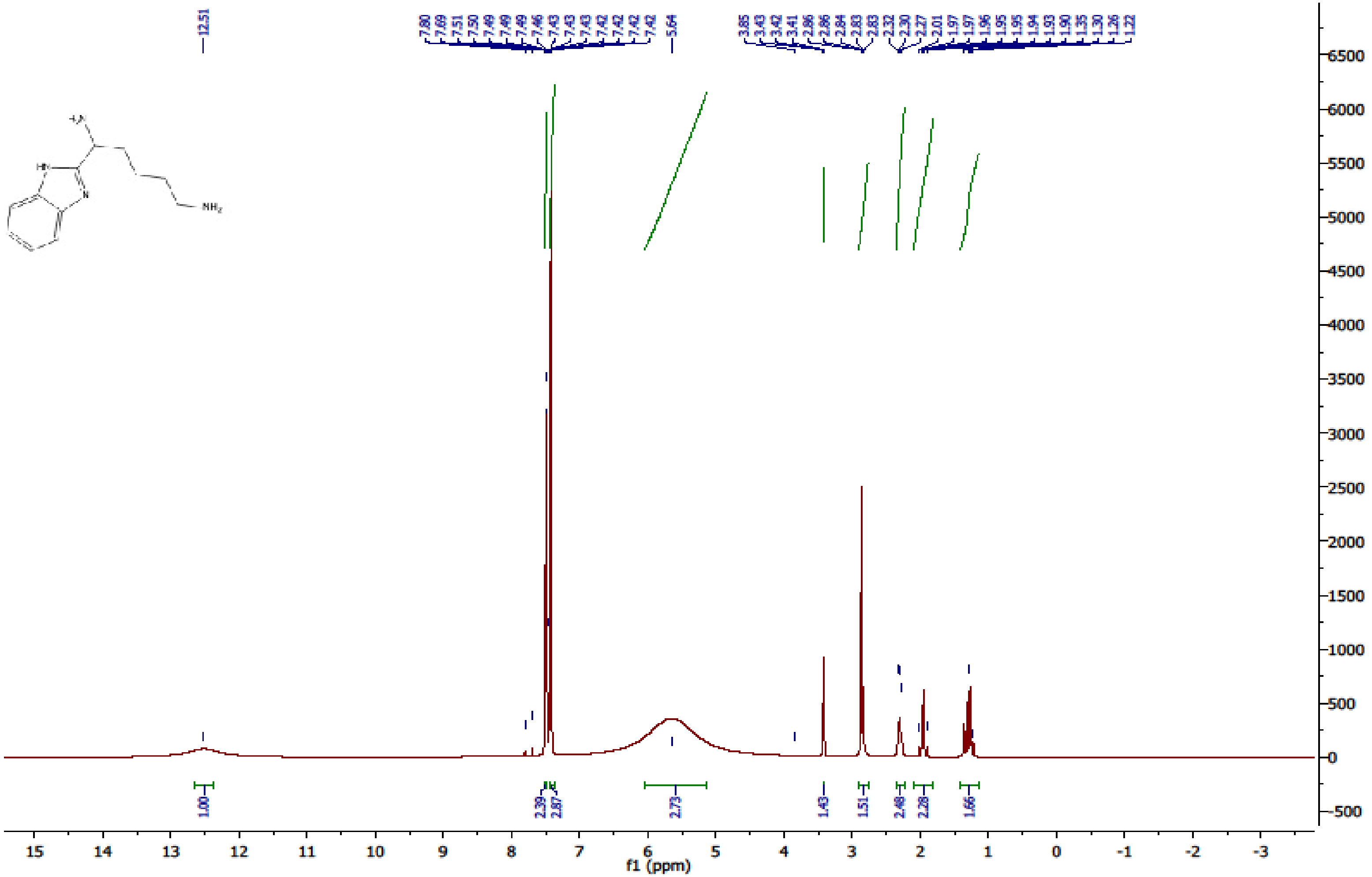

Figure S7: ^{1}H-NMR of 1-(1H-benzo[d]imidazol-2-yl) pentane-1, 5-diamine.

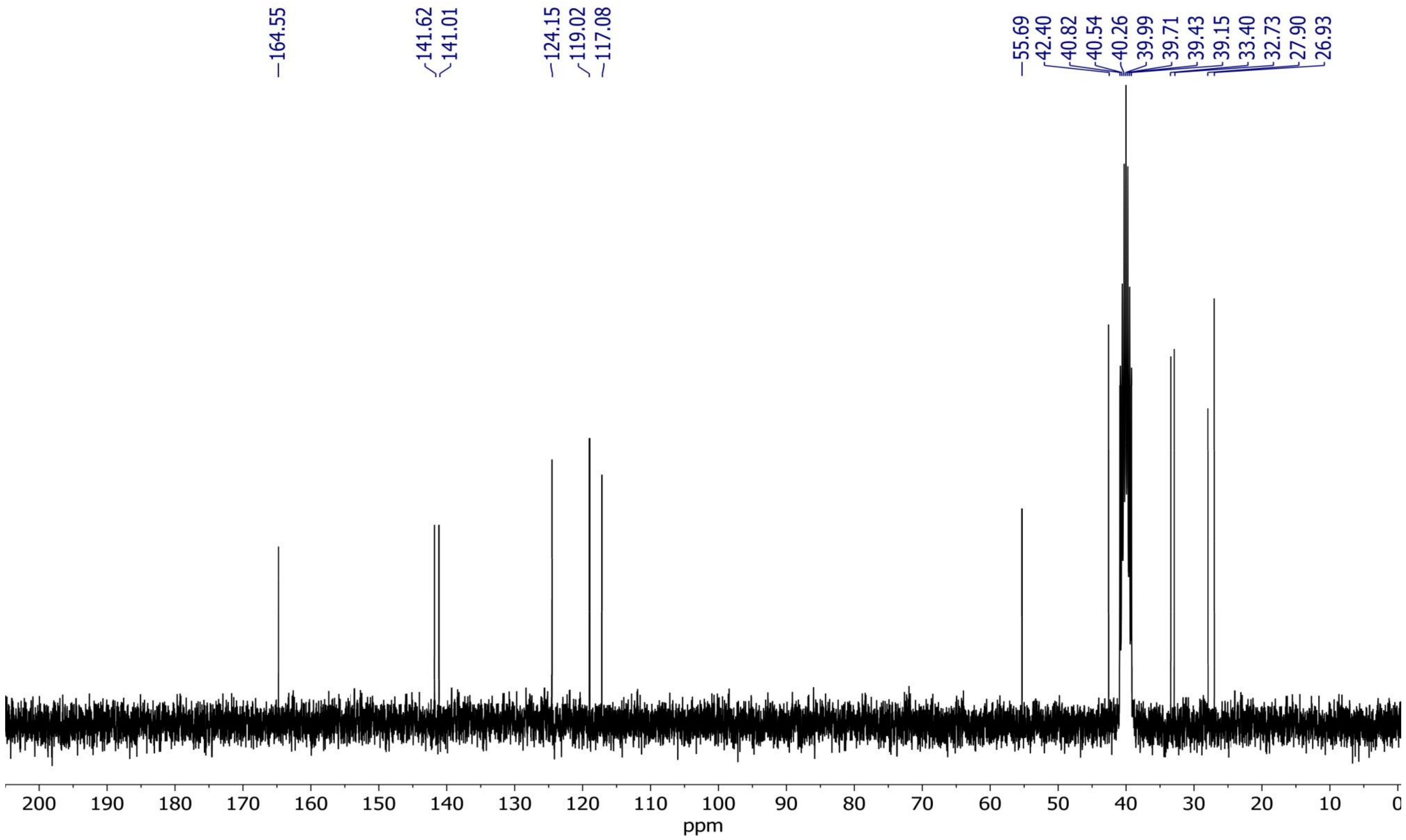

Figure S8: ^{13}C-NMR of (R)-1-(1H-benzo[d]imidazol-2-yl) pentane-1, 5-diamine.

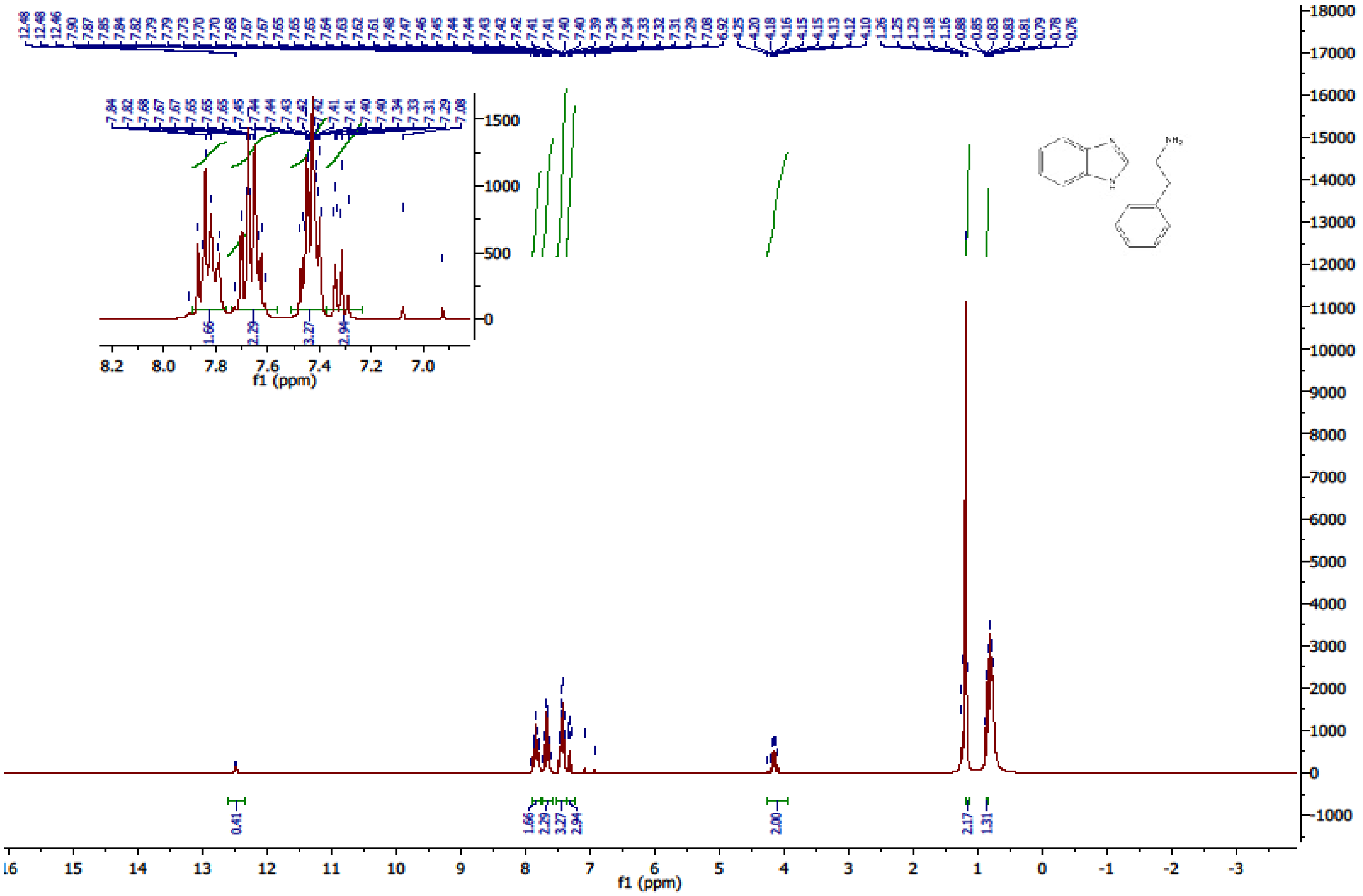

Figure S9: ^{1}H-NMR of (R)-1-(1H-benzo[d]imidazol-2-yl)-2-phenylethanamine.

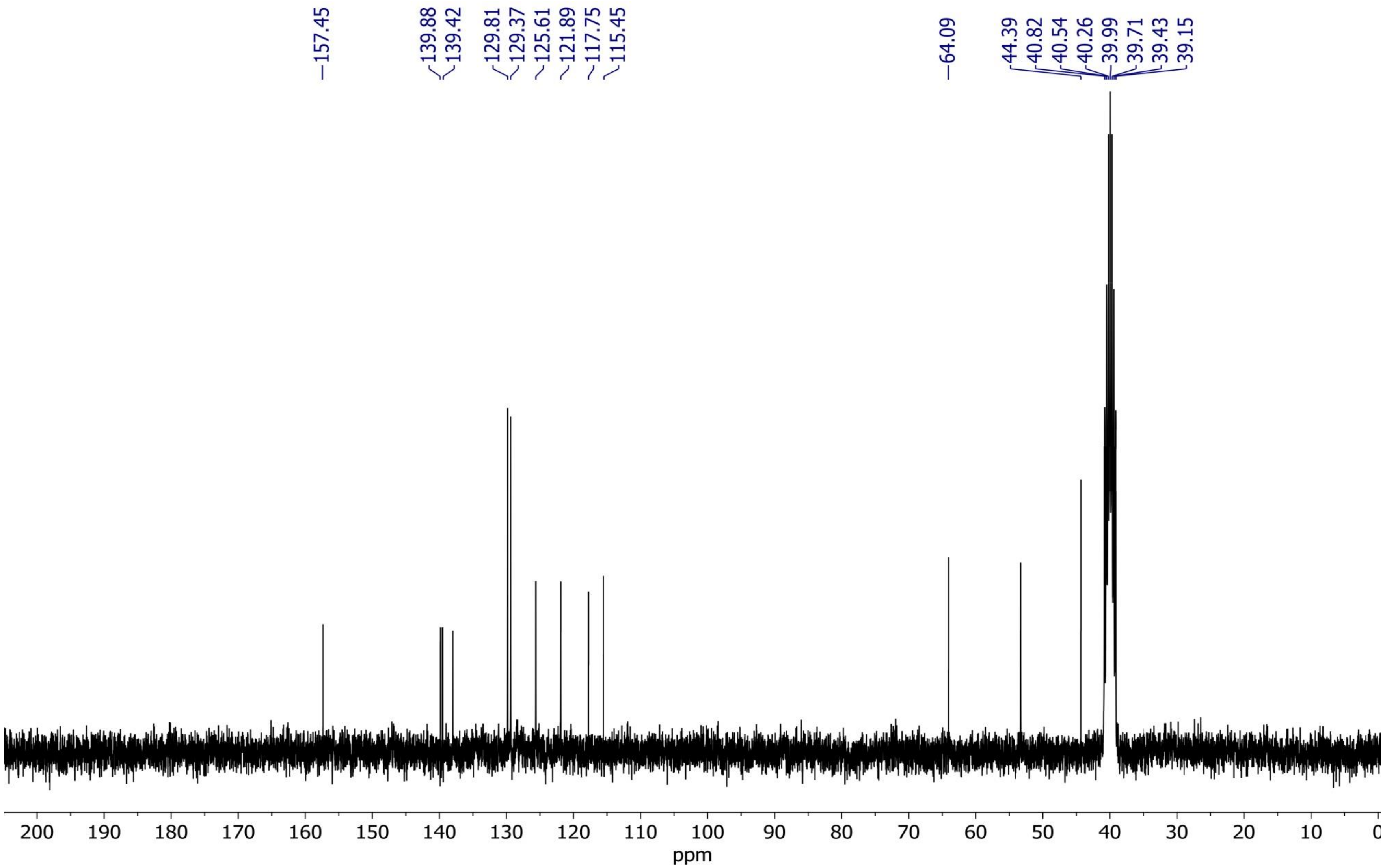

Figure S10: ^{13}C-NMR of (R)-1-(1H-benzo[d]imidazol-2-yl)-2-phenylethanamine.

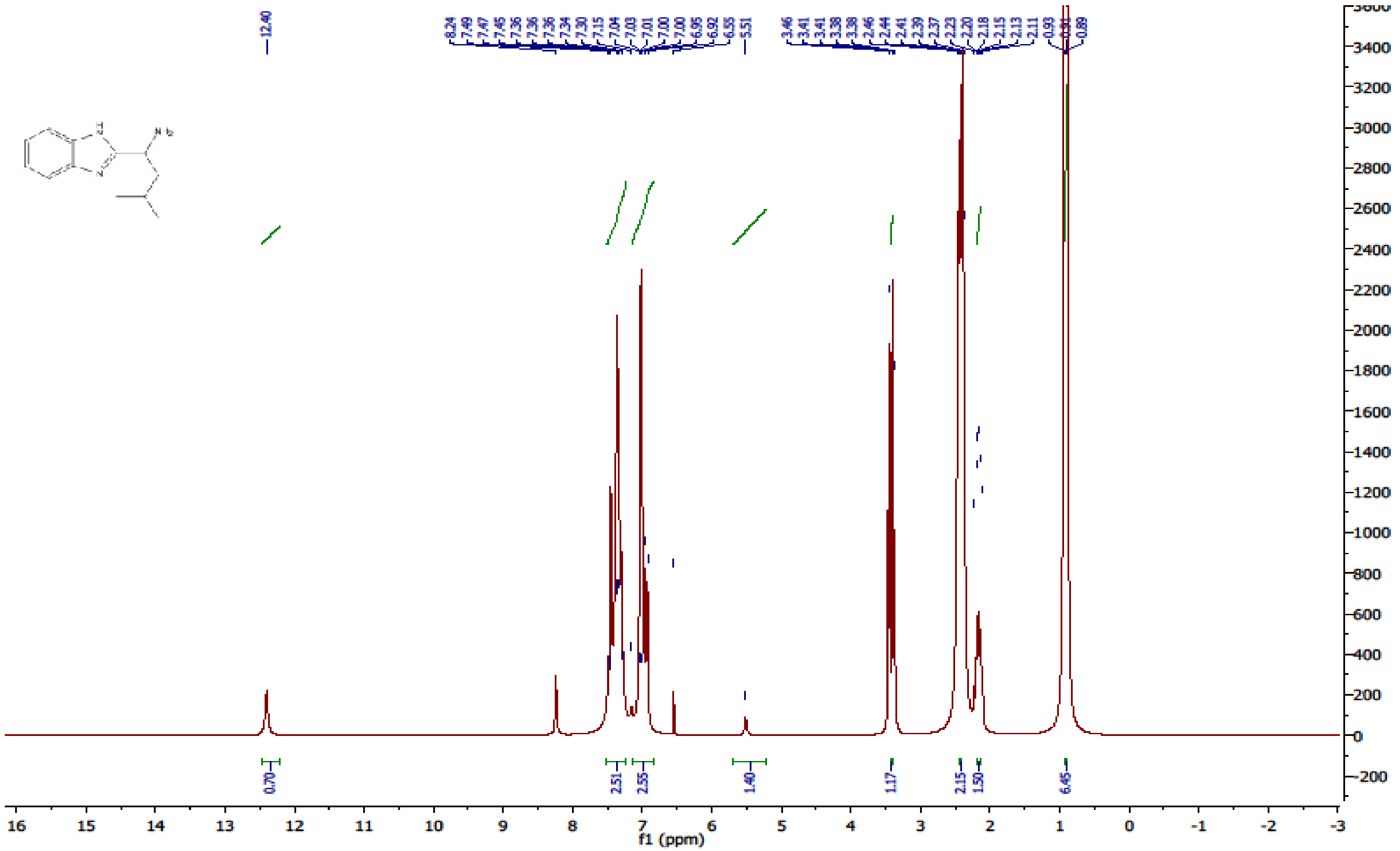

Figure S11: ^{1}H-NMR of (R)-1-(1H-benzo[d]imidazol-2-yl)-3-methylbutan-1-amine.

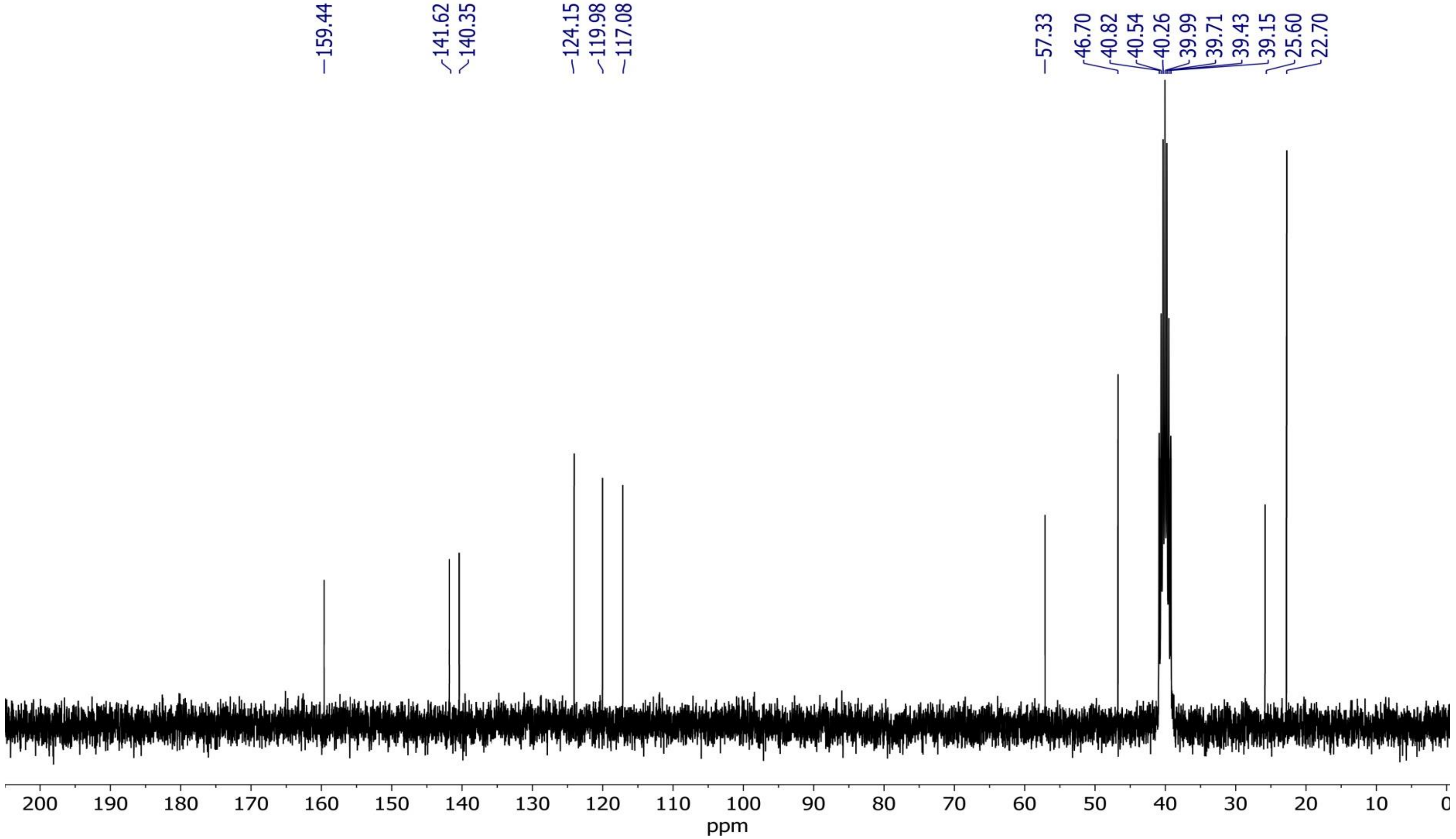

Figure S12: ^{13}C-NMR of (R)-1-(1H-benzo[d]imidazol-2-yl)-3-methylbutan-1-amine.

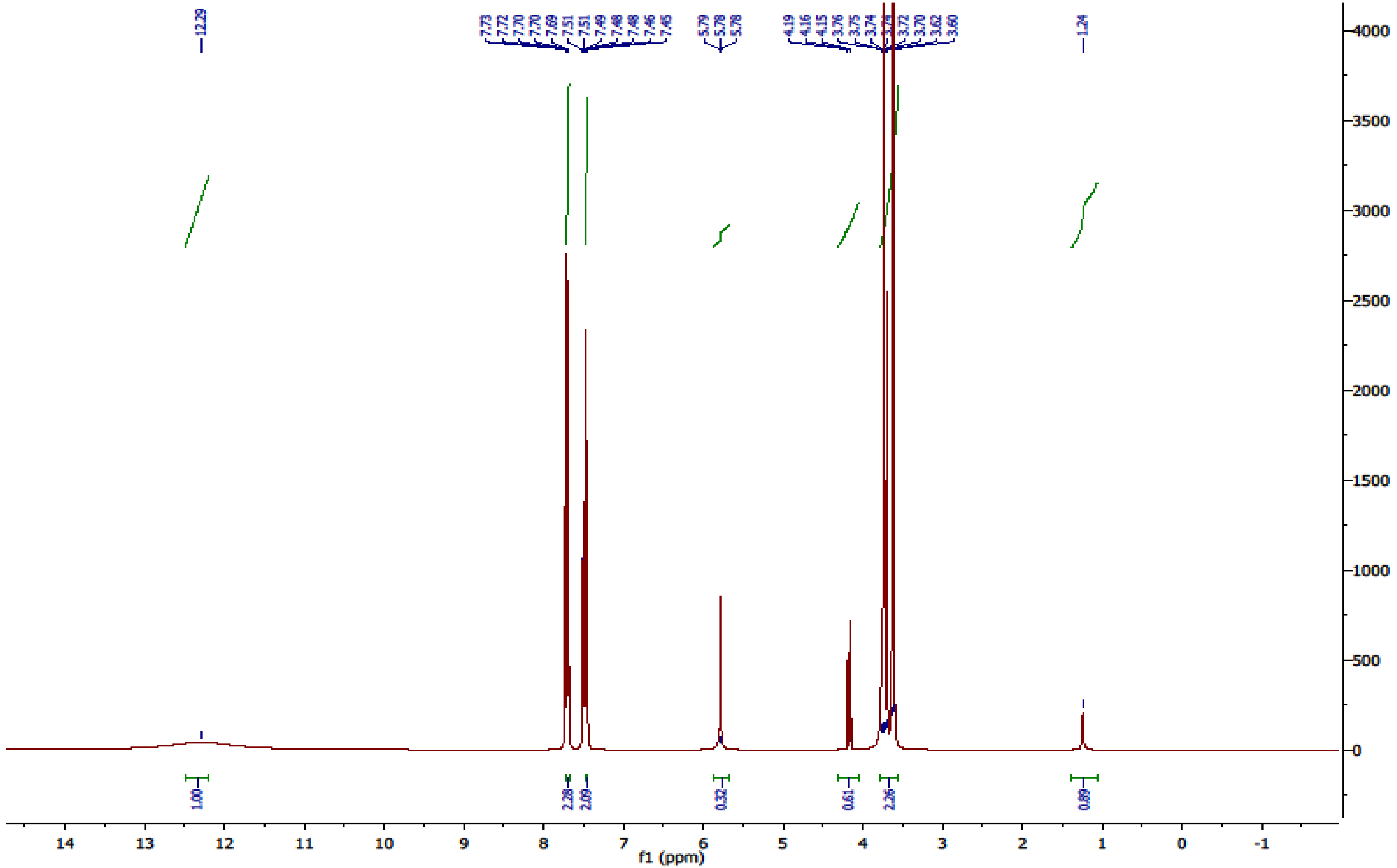

Figure S13: ^{1}H-NMR of (R)-2-amino-2-(1H-benzo[d]imidazol-2-yl) ethanethiol.

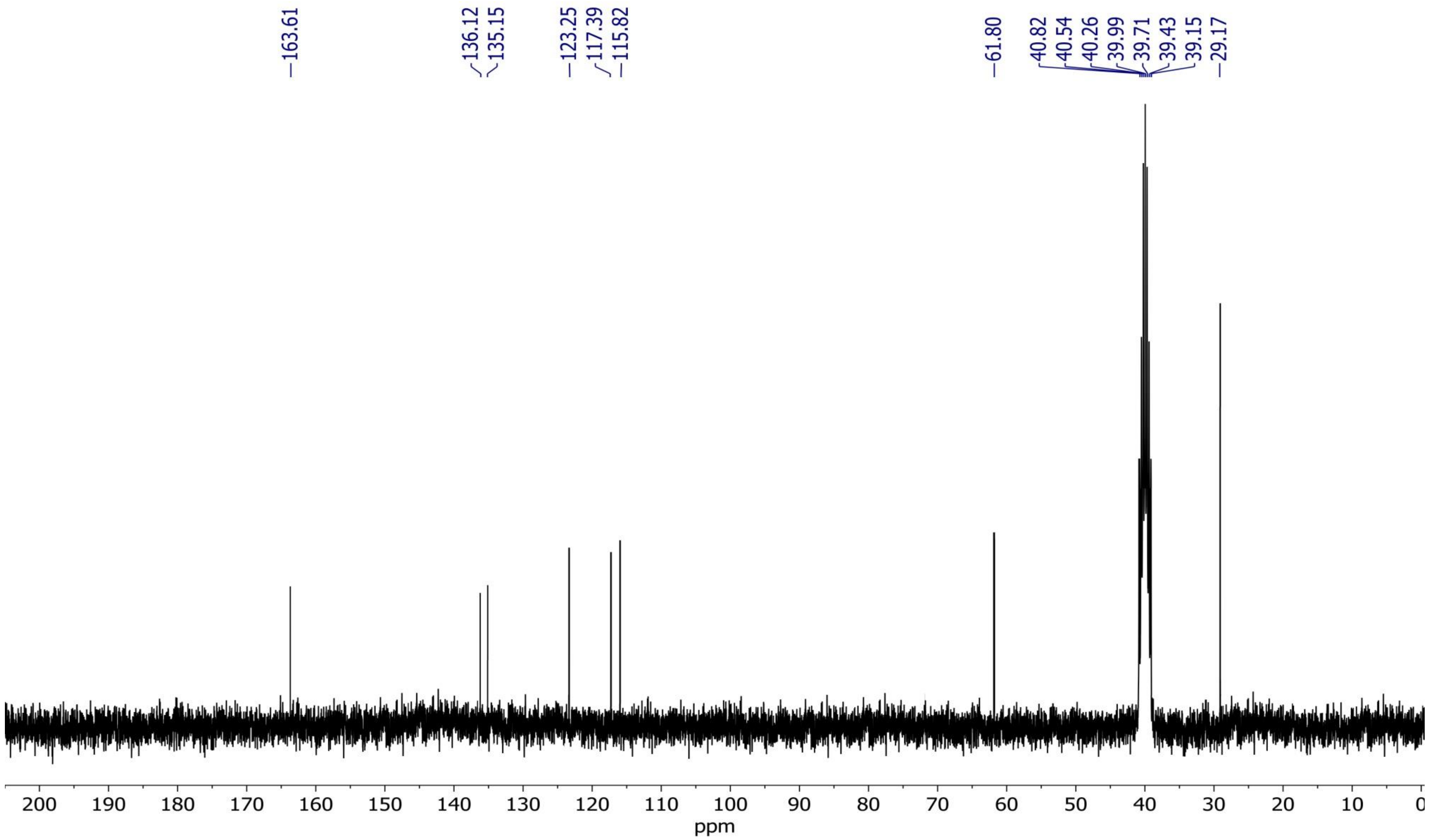

Figure S14: ^{13}C-NMR of (R)-2-amino-2-(1H-benzo[d]imidazol-2-yl) ethanethiol.

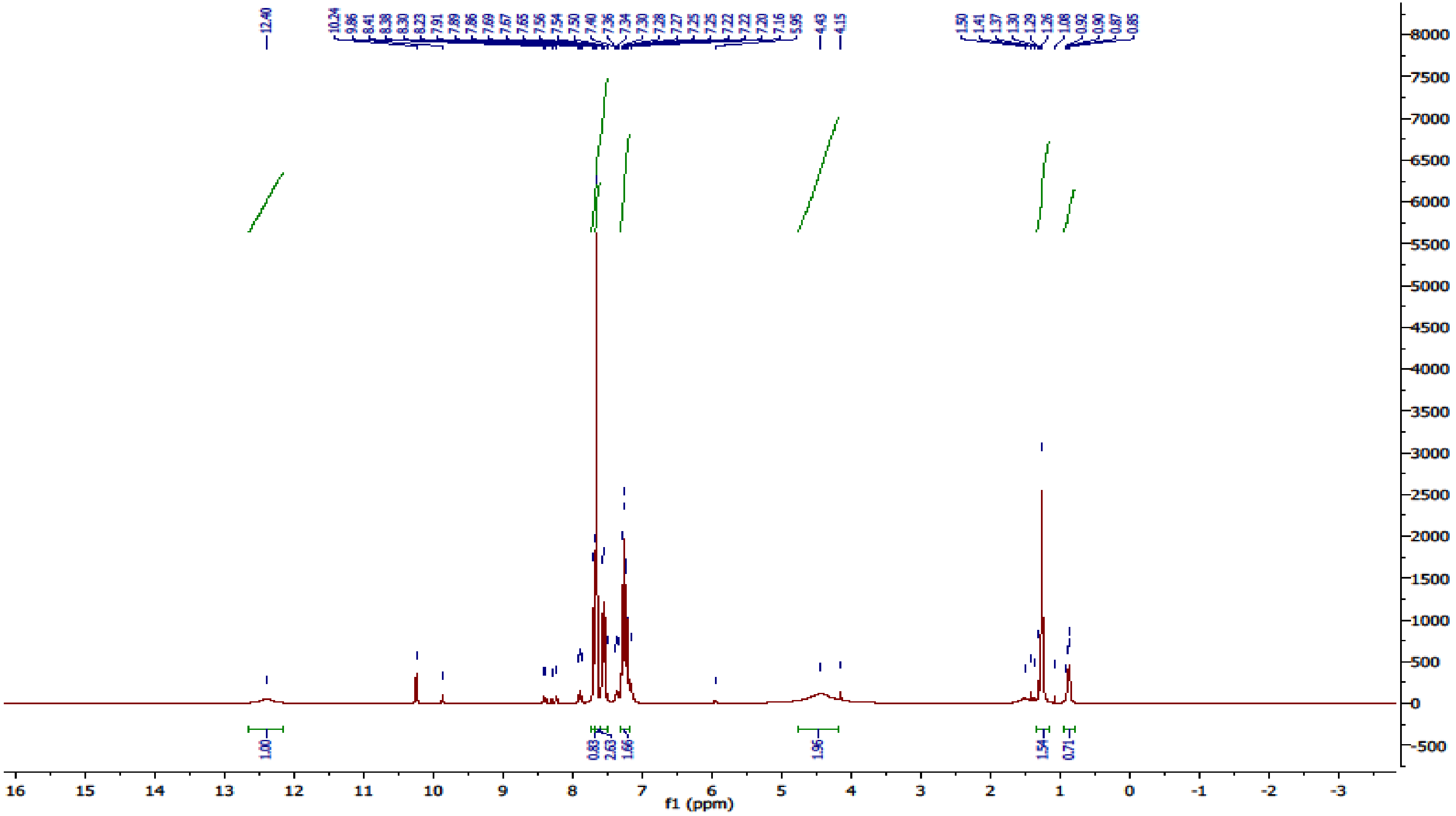

Figure S15: ^{1}H-NMR of (R)-3-amino-3-(1H-benzo[d]imidazol-2-yl) propanamide.

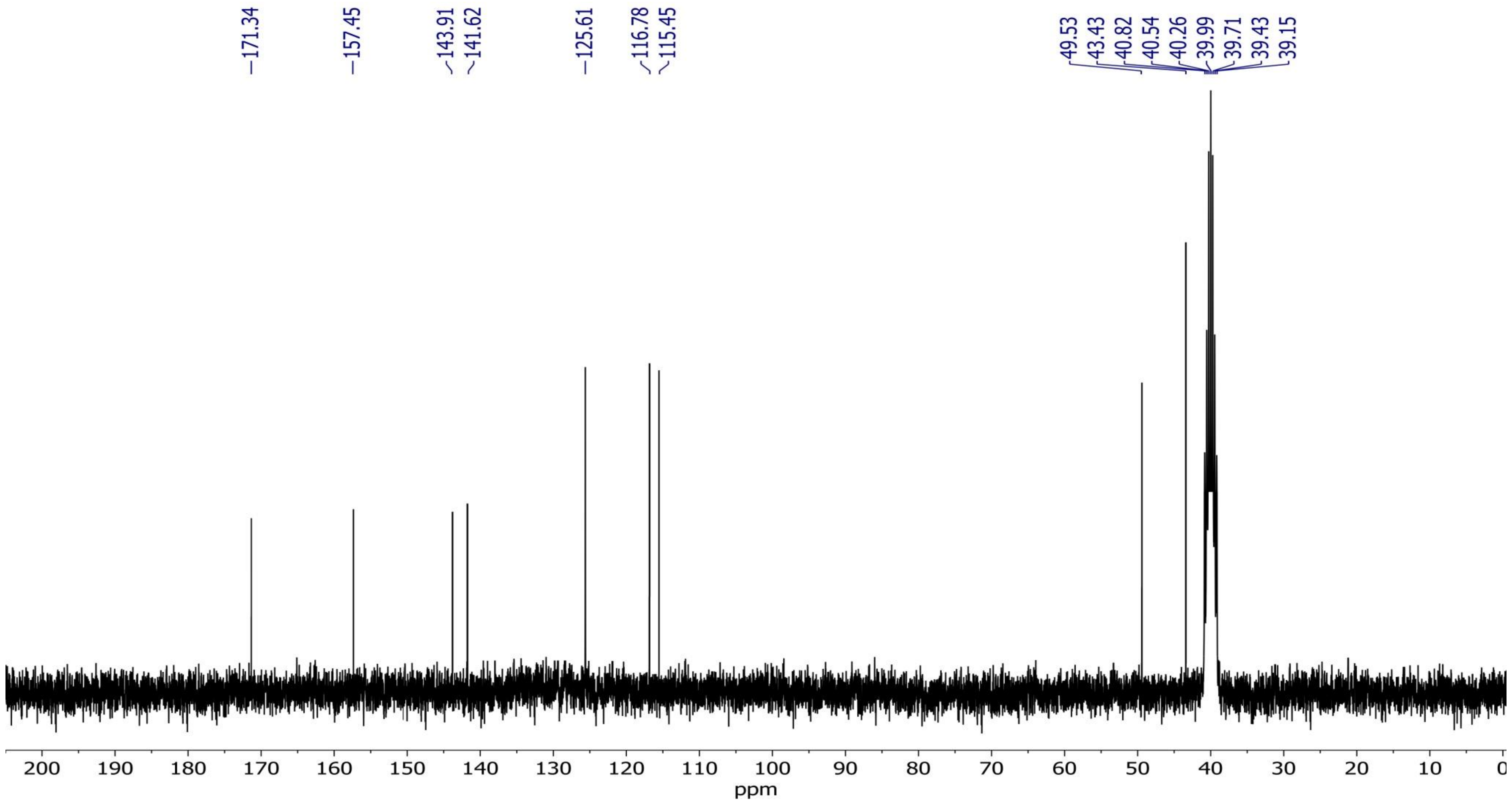

Figure S16: ^{13}C-NMR of (R)-3-amino-3-(1H-benzo[d]imidazol-2-yl) propanamide.

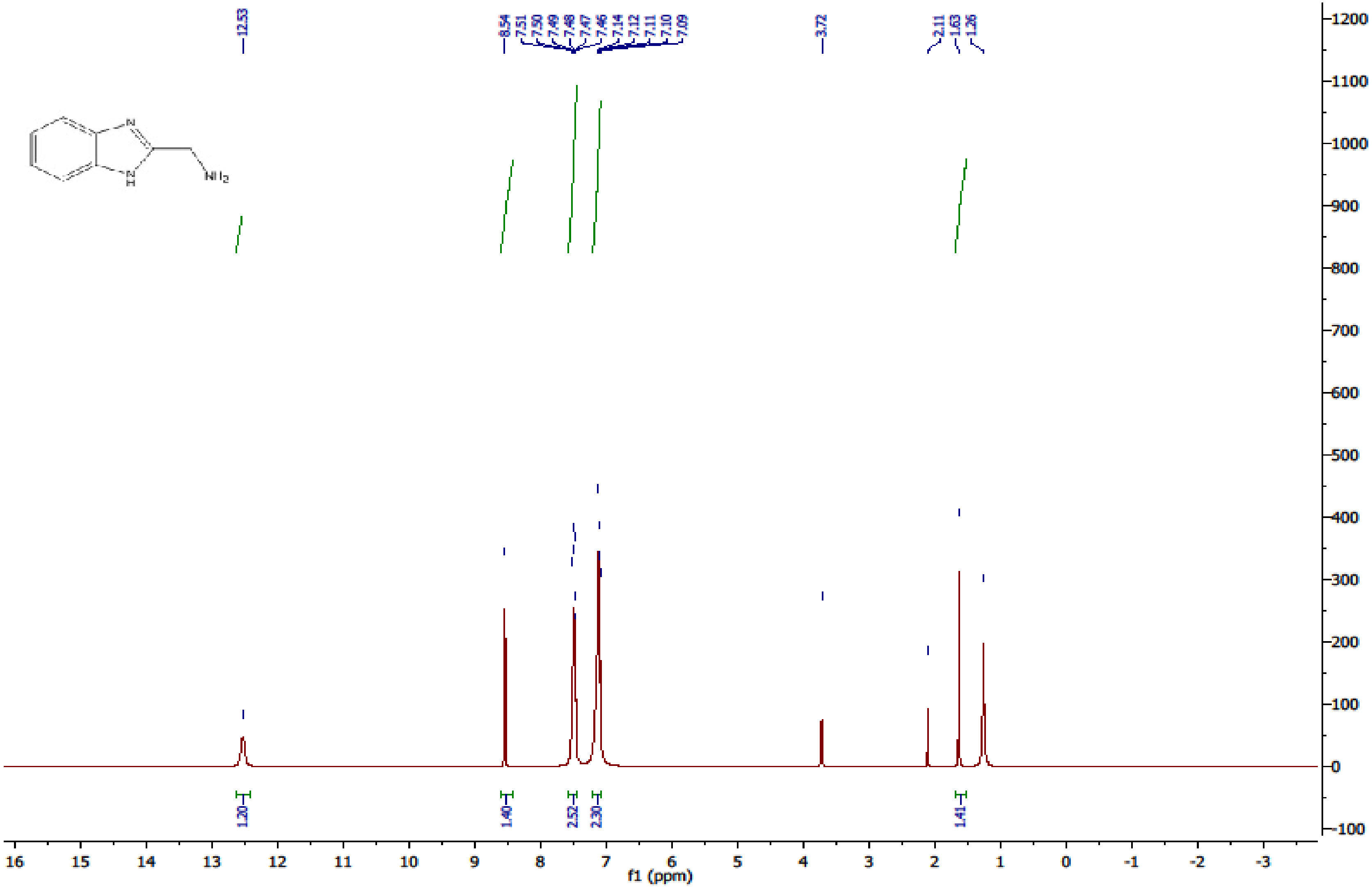

Figure S17: ^{1}H-NMR of (1H-benzo[d]imidazol-2-yl) methanamine.

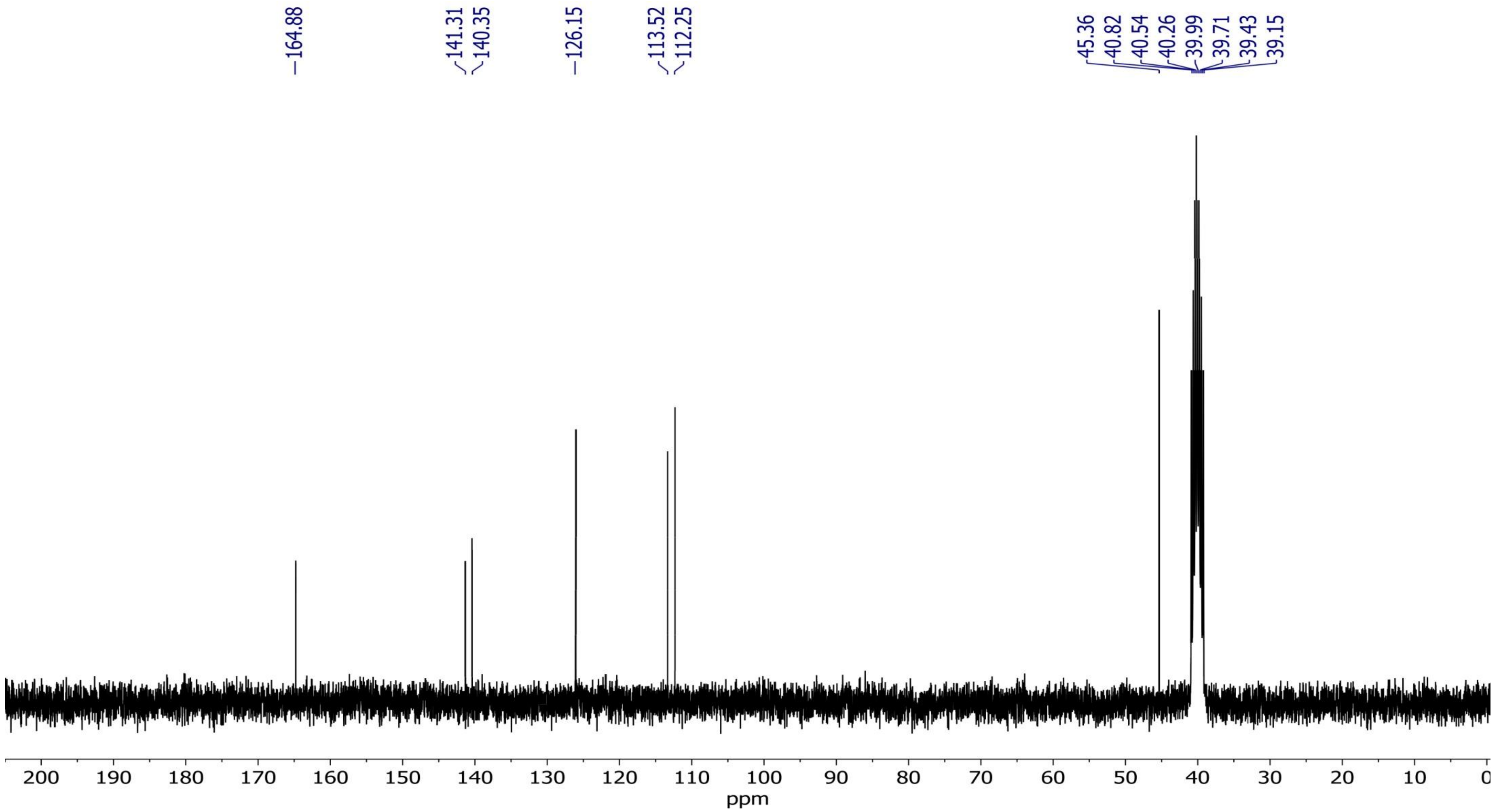

Figure S18: ^{13}C-NMR of (1H-benzo[d]imidazol-2-yl) methanamine.

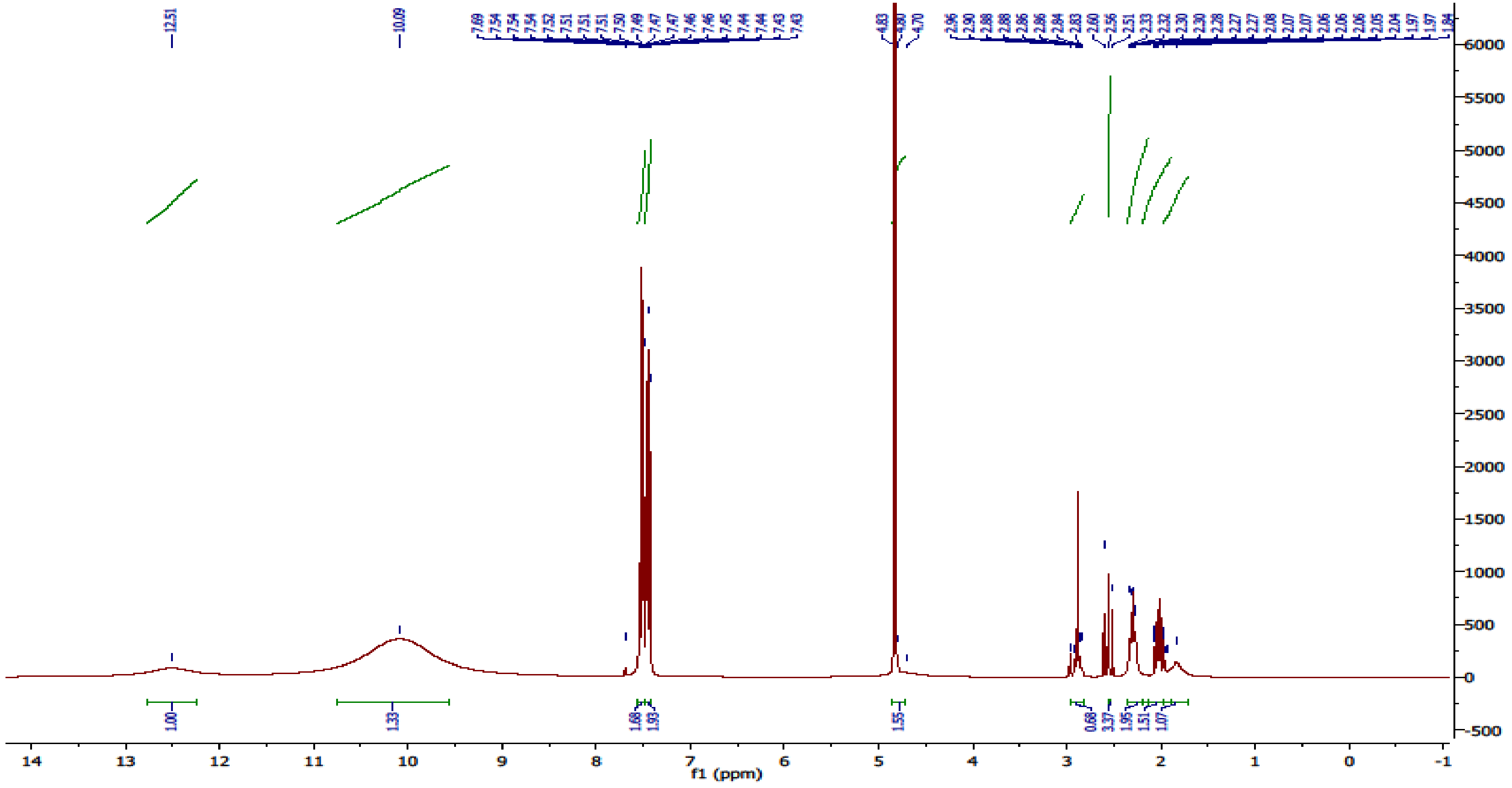

Figure S19: ^{1}H-NMR of (R)-amino ((4-amino-4-(1H-benzo[d]imidazol-2-yl) butyl) amino) methaniminium.

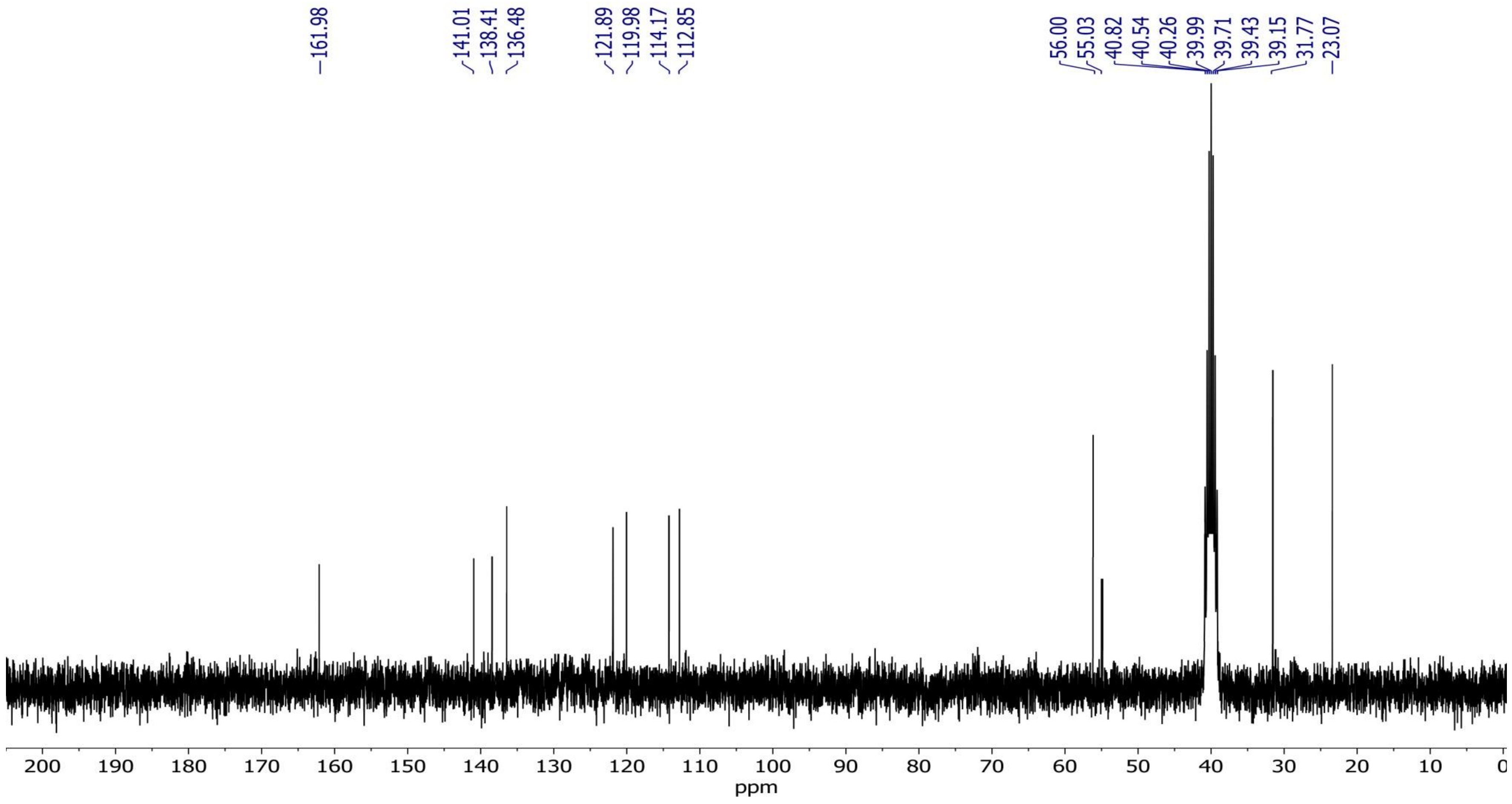

Figure S20: ^{13}C-NMR of (R)-amino ((4-amino-4-(1H-benzo[d]imidazol-2-yl) butyl) amino) methaniminium.

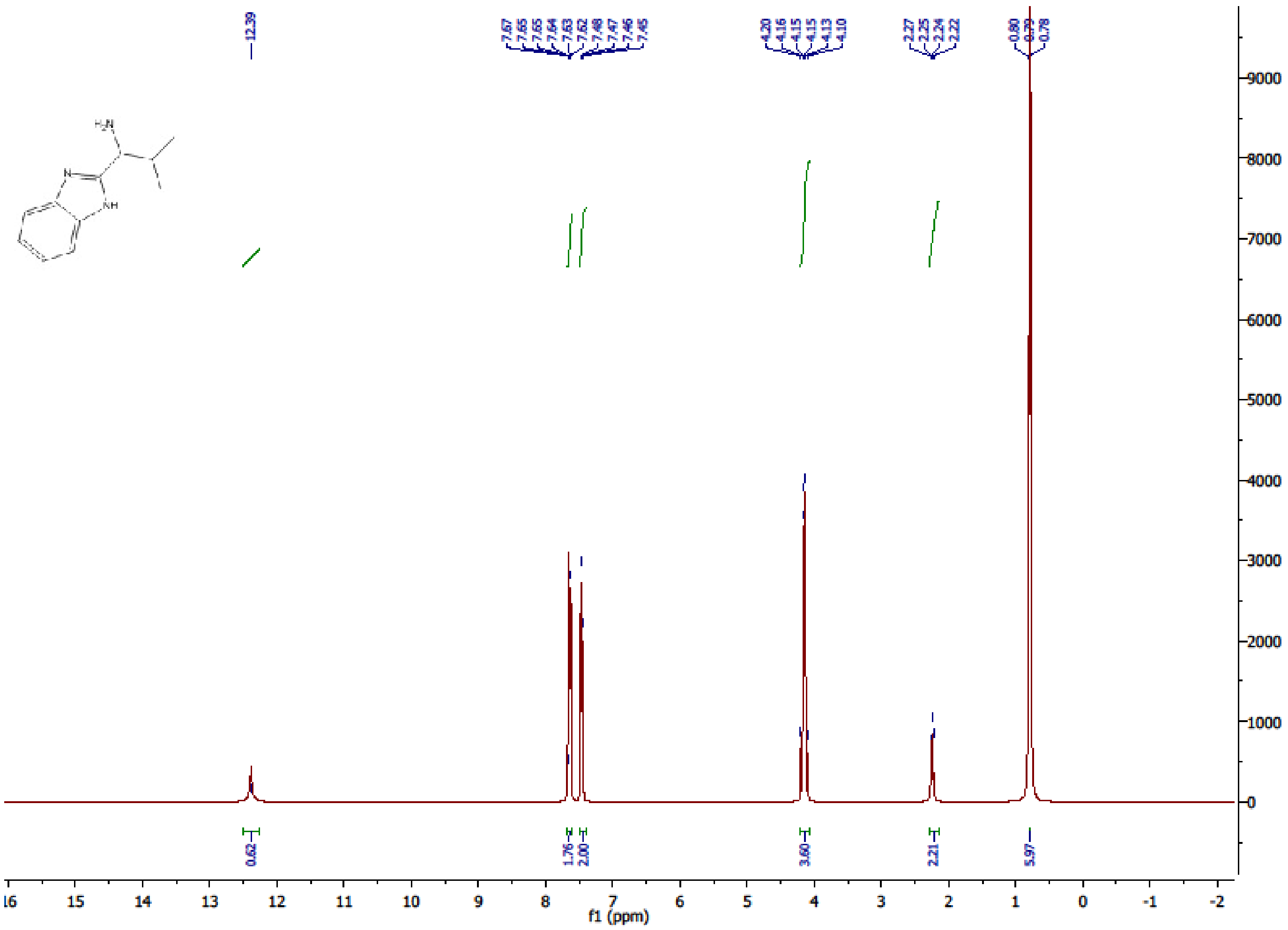

Figure S21: ^{1}H-NMR of (R)-1-(1H-benzo[d]imidazol-2-yl)-2-methylpropan-1-amine.

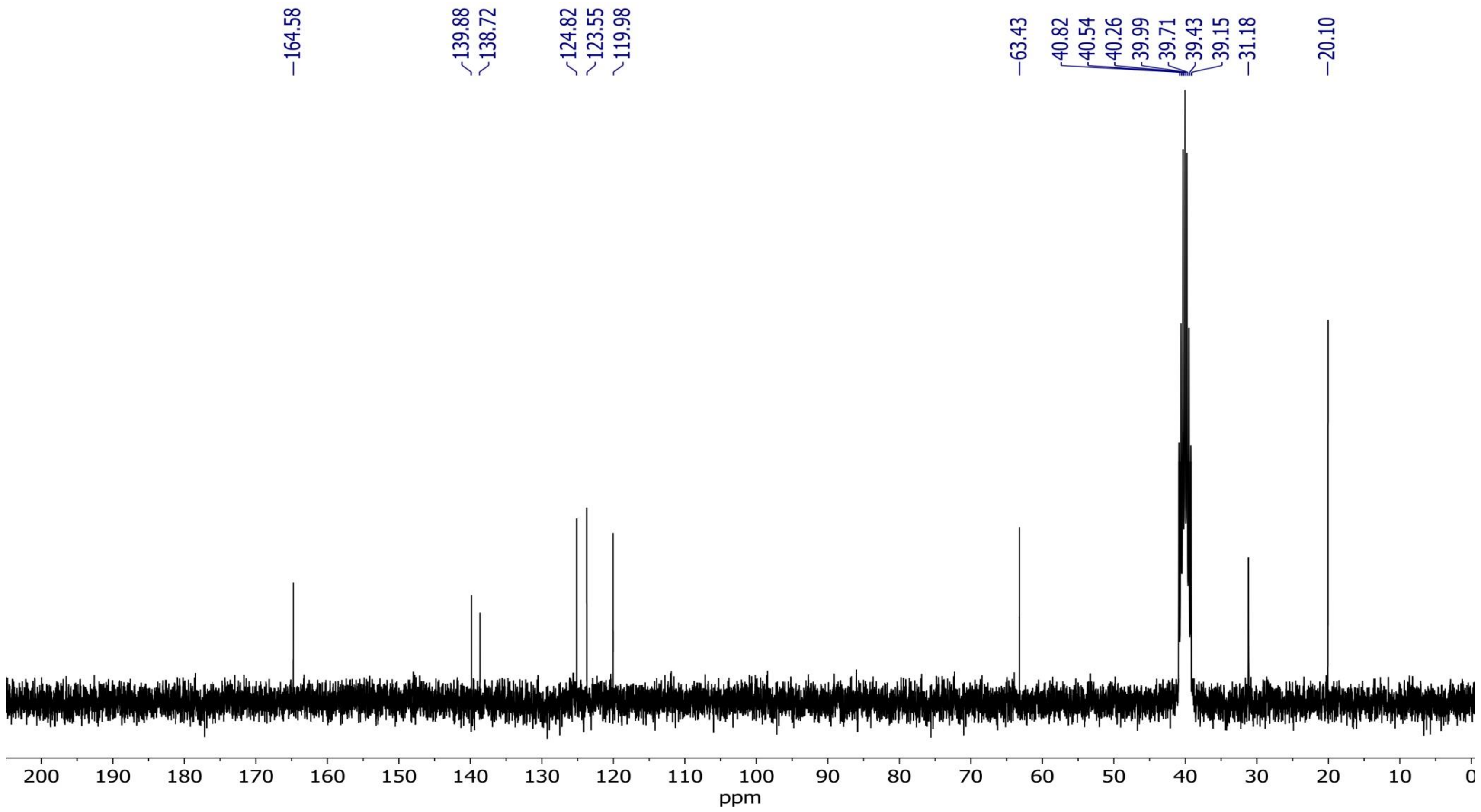

Figure S22: ^{13}C-NMR of (R)-1-(1H-benzo[d]imidazol-2-yl)-2-methylpropan-1-amine.

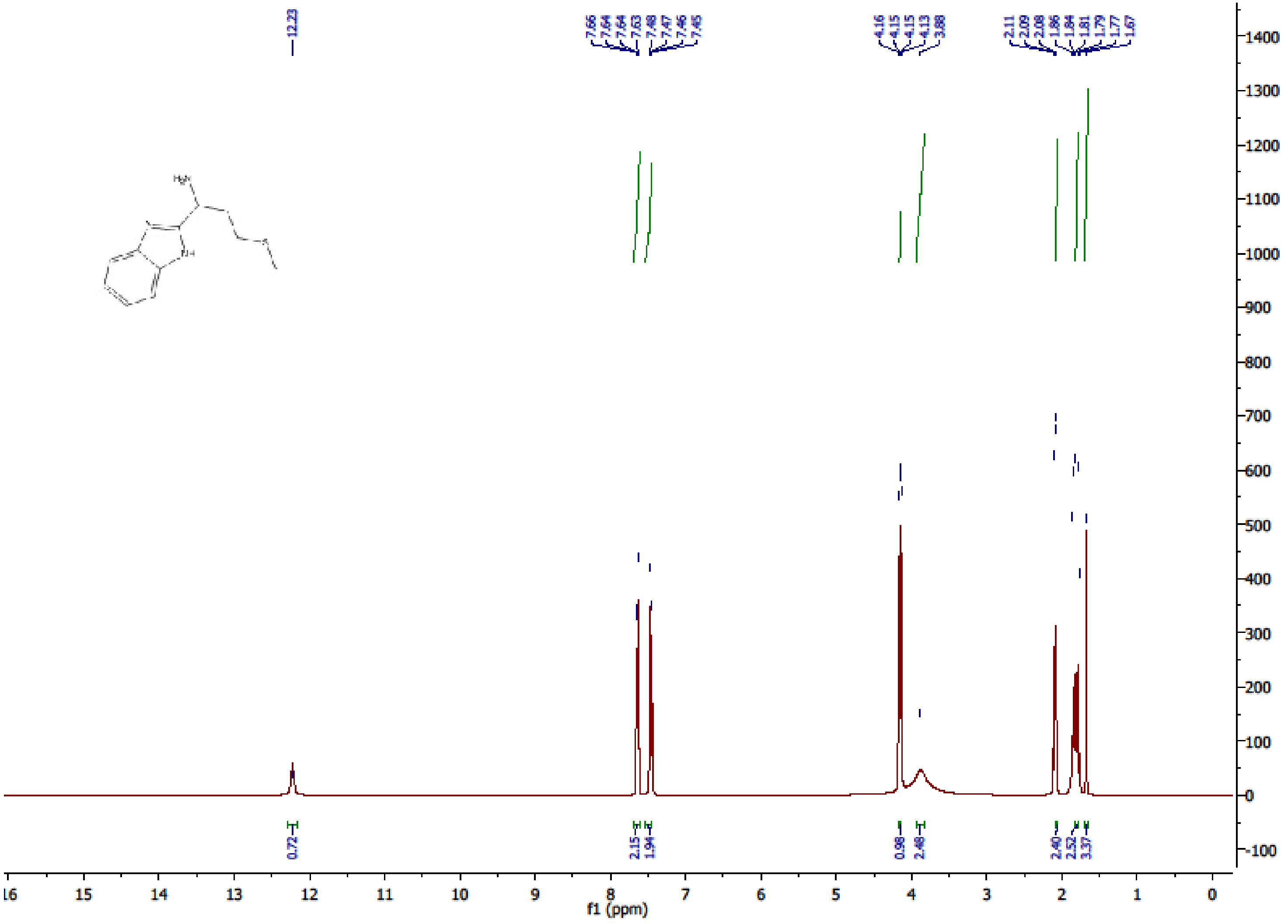

Figure S23: ^{1}H-NMR of (R)-1-(1H-benzo[d]imidazol-2-yl)-3-(methylthio) propan-1-amine.

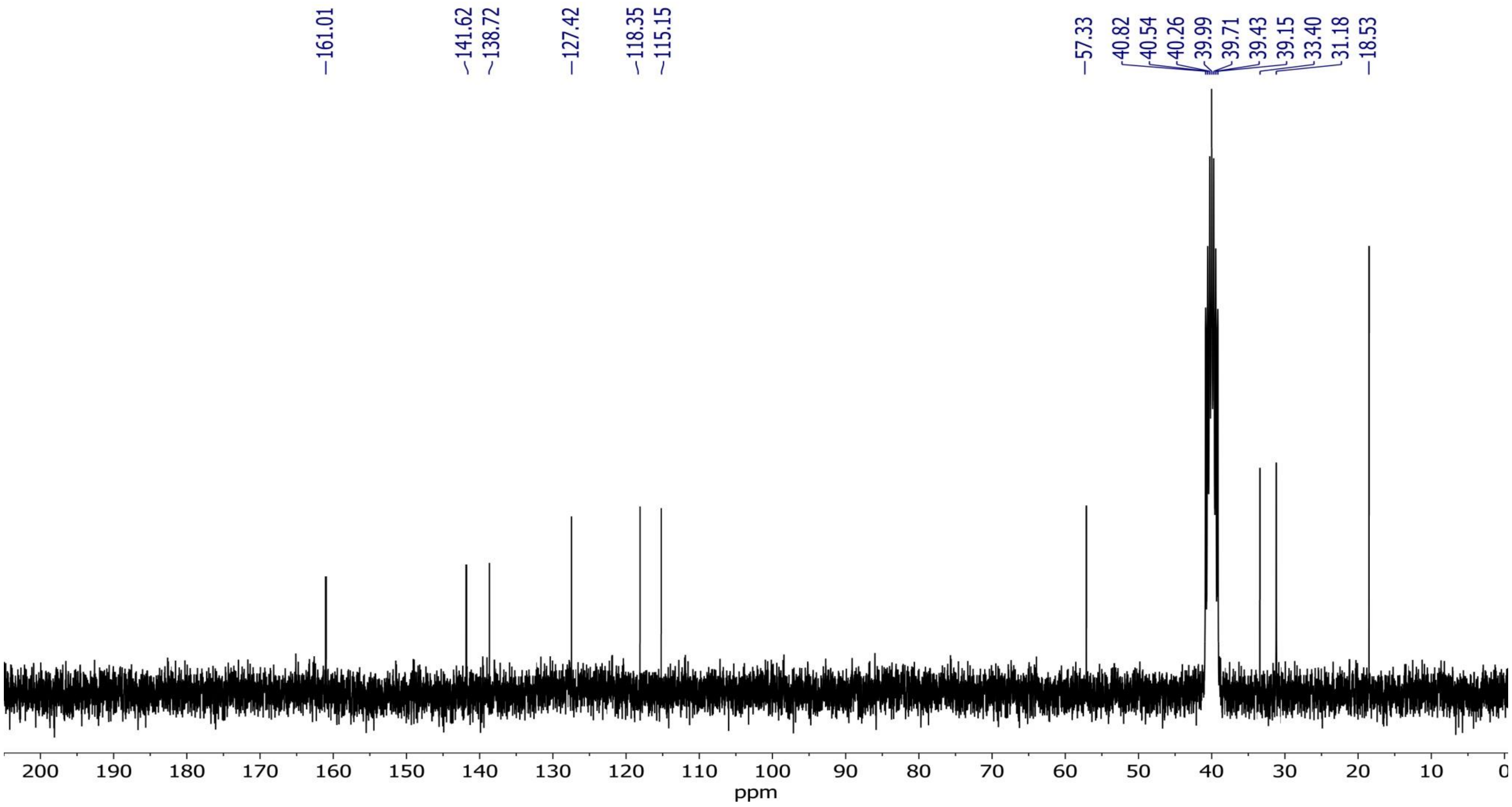

Figure S24: ^{13}C-NMR of (R)-1-(1H-benzo[d]imidazol-2-yl)-3-(methylthio) propan-1-amine.

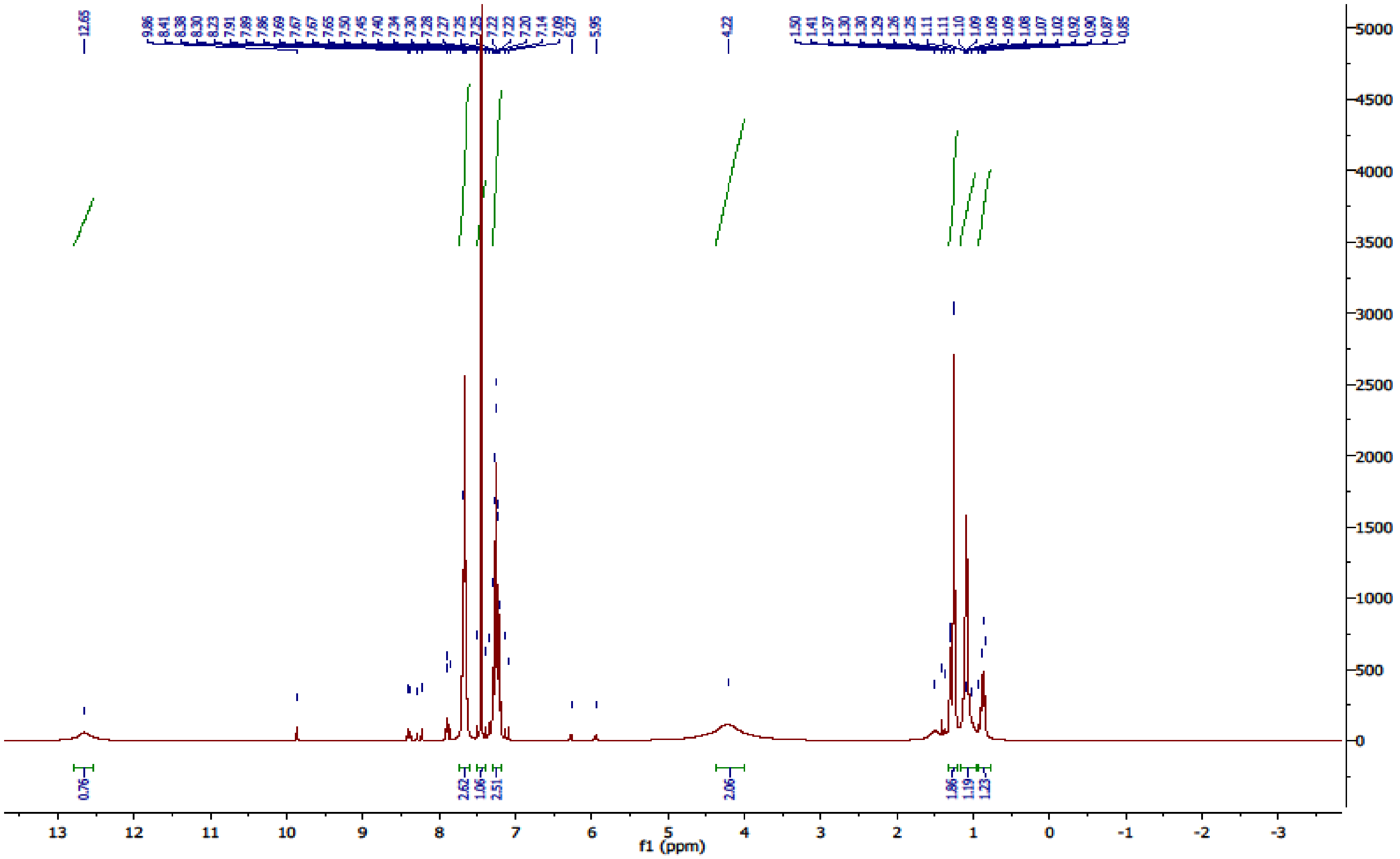

Figure S23: ^{1}H-NMR of (R)-4-amino-4-(1H-benzo[d]imidazol-2-yl) butanamide.

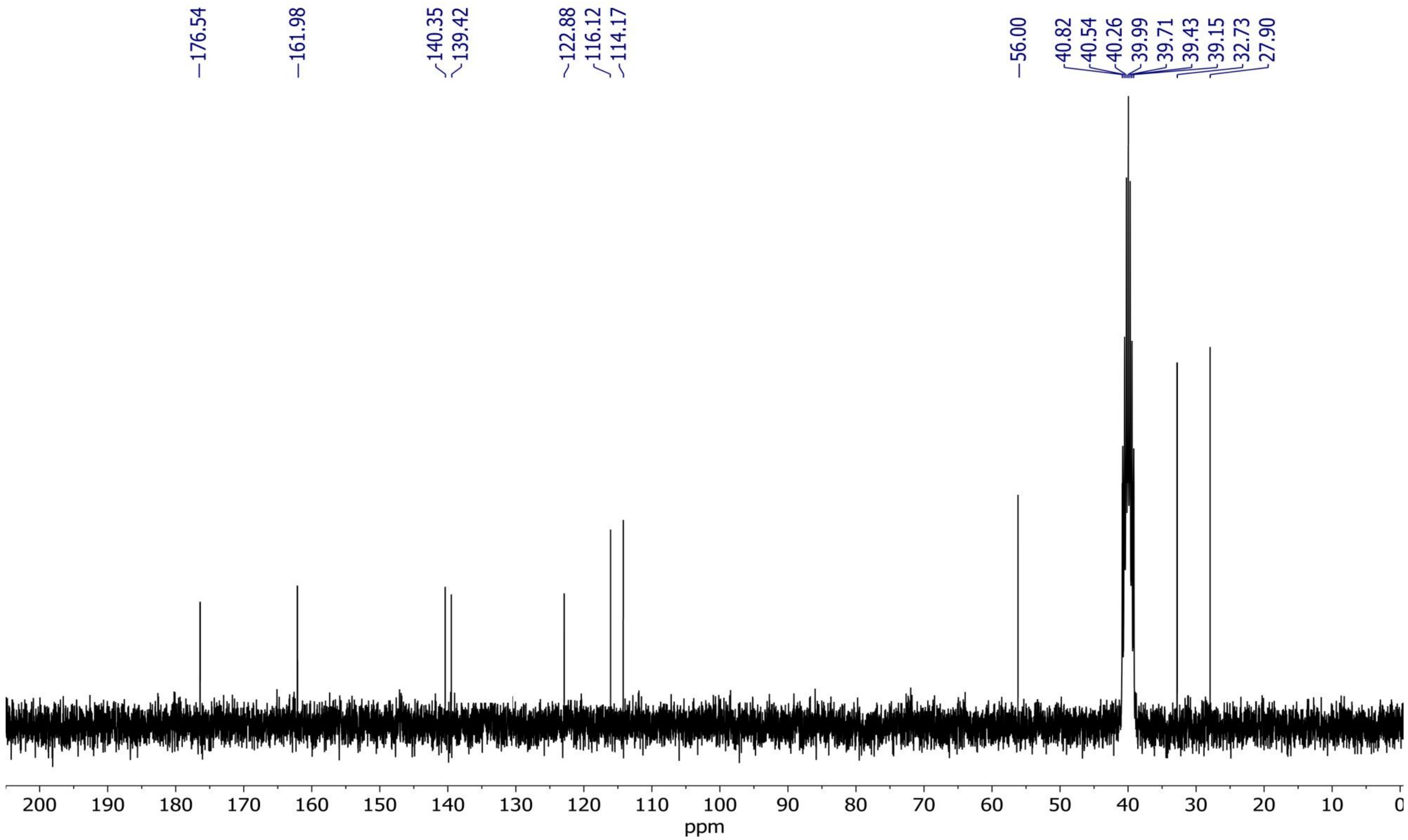

Figure S24: ^{13}C-NMR of (R)-4-amino-4-(1H-benzo[d]imidazol-2-yl) butanamide.
